# CircMETTL3-156aa reshapes the glycolytic metabolism of macrophages to promote M1 polarization and induce cytokine storms in sHLH

**DOI:** 10.1038/s41420-024-02202-0

**Published:** 2024-10-09

**Authors:** Longlong Xie, Xiangying Deng, Xiao Li, Xun Li, Xiangyu Wang, Haipeng Yan, Lin Zhao, Dan Yang, Ting Luo, Yufan Yang, Zhenghui Xiao, Xiulan Lu

**Affiliations:** 1 Department of Radiology, Hunan Provincial Key Laboratory of Pediatric Orthopedics, The Affiliated Children’s Hospital of Xiangya School of Medicine, Central South University (Hunan children’s hospital), Changsha, Hunan China; 2 Department of Pediatric Intensive Care Unit (PICU) and Hunan Provincial Key Laboratory of Emergency Medicine for Children, The Affiliated Children’s Hospital of Xiangya School of Medicine, Central South University (Hunan children’s hospital), Changsha, Hunan China; 3 Institute of Medical Sciences, National Clinical Research Center for Geriatric Disorders, Xiangya Hospital, Central South University, Changsha, Hunan China; 4 Hengyang Medical College, University of South China, Hengyang, Hunan China; 5 Pediatrics Research Institute of Hunan, The Affiliated Children’s Hospital of Xiangya School of Medicine, Central South University (Hunan children’s hospital), Changsha, Hunan China; 6 Department of Pathology, The Second Xiangya Hospital, Central South University, Changsha, Hunan China

**Keywords:** Immunology, Non-coding RNAs, Cell signalling

## Abstract

Persistent macrophage activation and cytokine storms are critical causes for the rapid disease progression and high mortality rate of Secondary Hemophagocytic lymphohistiocytosis (sHLH). Identification of key regulatory factors that govern the activation of macrophages is vital. Plasma exosomal circular RNAs (circRNAs) are considered important biomarkers and potential therapeutic targets for various diseases, however, their function in sHLH is still unclear. In this study, we demonstrated for the first time that circMETTL3, derived from METTL3, is upregulated in sHLH patient plasma exosomes, which may plays an important role in the diagnosis of sHLH. Significantly, we also revealed that a novel peptide encoded by circMETTL3, METTL3-156aa, is an inducer of M1 macrophage polarization, which is responsible for the development of cytokine storms during sHLH. We then identified that METTL3-156aa binding with lactate dehydrogenase A (LDHA) and promotes M1 macrophage polarization by enhancing macrophage glycolysis. Additionally, the glycolysis metabolite lactate upregulates the cleavage factor SRSF10 expression by lactylation. This results in increased splicing of the pre-METTL3 mRNA, leading to an enchance in the production of cirMETTL3. Therefore, our results suggest that the *circMETTL3/METTL3-156aa/LDHA/Lactate/SRSF10* axis forms a positive feedback loop and may be a novel therapeutic target for the treatment of sHLH.

## Introduction

Hemophagocytic syndrome (HPS), also known as hemophagocytic lymphohistiocytosis (HLH), is a rare and fatal childhood disease characterized by multiple organ damage induced by overactivation of the immune system [1]. HLH usually occurs as a result of inherited genetic defects or acquired risk factors, including autoimmune disorders, malignant neoplasms or viral infections [2]. Secondary hemophagocytic syndrome (sHLH) is more common in children [3, 4]. In the forms of pediatric sHLH, hyperproliferation of activated macrophages and T cells leads to the excessive release of cytokines, contributing to an typical clinical profile dominated by fever, pancytopenia, hypotension and tissue inflammation progresses rapidly and has a high mortality rate, making early recognition and treatment more challenging [5]. Central to the mechanisms of sHLH pathogenesis is a positive feedback loop of sustained macrophage activation and cytokine storms with cytokines including interferon (IFN-γ), interleukins IL-1β, IL-2, IL-6, IL-12, IL-18, and the tumor necrosis factor TNF-ɑ [6, 7]. Therefore, new insights into the pathogenesis and progression mechanisms of sHLH could assist in the clinical search for early diagnostic markers of sHLH and the exploration of precise therapeutic strategies.

Macrophage activation is a form of HLH [8] that plays a central role in cytokine storms. Generally, macrophages are mainly categorized into the classically activated inflammatory type (M1) and selectively activated anti-inflammatory type with immunosuppressive function (M2), which regulate different cytokines. M1 macrophages exhibit proinflammatory functions and secrete inflammatory cytokines such as IL-6, iNOS, TNF-α, IL-18 and IL-1β and chemokines CXCL9 and CXCL10, while M2 macrophages secrete high levels of anti-inflammatory cytokines, including ARG1, IL-10 and TGF-β [9]. Macrophage function is closely related to cellular metabolism. Glucose metabolism is not only an important channel for cellular nutrient supply, but also an important regulator of macrophage activation and function [10]. Aerobic glycolysis was found to be one of the most prominent metabolic features of classically activated macrophage M1 type. Glycolysis produces pyruvate, which enters the tricarboxylic acid cycle and increases cytoplasmic levels of Acetyl-coenzyme A (Acetyl-CoA) and histone acetylation modifications through the production of citrate, which in turn promotes the transcription of proinflammatory cytokines in M1 macrophages [11]. Lactate is the end product of glycolysis and regulates inflammatory responses in various cells. At the same time, during inflammation, immune cells produce and secrete large amounts of lactate, further amplifying the pro-inflammatory phenotype [12]. Nevertheless, mechanistic studies elucidating sHLH macrophage activation from a metabolic perspective are not well understood.

Circular RNAs (circRNAs) are a special class of first- and tail-linked noncoding RNAs with a closed-loop structure formed by the reverse splicing of exons or introns during the splicing of precursor mRNAs. It has no 5’ cap or 3’ polyadenylated tail structure and is more stable than linear mRNAs, with high abundance and tissue specificity [13]. CircRNAs can modulate the pathophysiological processes of disease by working with RNA [14, 15], DNA [16], proteins [17] or encoding small peptides [18] to engage in intracellular signaling. sHLH manifests as an imbalance in immune system homeostasis with excessive proliferation of activated macrophages and T cells. Recently, the functions of some circular RNAs in immune-related diseases have been reported [19, 20]. Hsa_circ_0002715 and Hsa_circ_0035197 in peripheral blood have been used as diagnostic and prognostic markers for rheumatoid arthritis [21]. Hsa_circ_0082688-hsa_circ_0008675 can predict the progression of systemic lupus erythematosus disease [22]. However, there are no reports on the function and mechanism of circRNA-induced immune responses during the development and progression of sHLH. In this study, we analyzed the expression profile of circRNAs in sHLH patients and found that is highly expressed in the plasma of patients with sHLH, which suggests that circMETTL3 may provide new references for the diagnosis of sHLH. Furthermore, we revealed that circMETTL3 in plasma exosomes induced M1 macrophage polarization in vitro and in vivo in sHLH models. We identified the molecular mechanism by which circMETTL3 enhances macrophage glycolysis by encoding the polypeptide METTL3-156aa, which binds to LDHA. Additionally, the glycolysis metabolite lactate increases the cleavage factor SRSF10 expression by inducing histone lactylation at the SFSF10 gene promoter. Then, cleavage of pre-METTL3 mRNA leads to increased production of cirMETTL3. Overall, the *circMETTL3/METTL3-156aa/LDHA/LA/SRSF10* axis forms a positive feedback loop and may be a novel therapeutic target for the treatment of sHLH.

## Results

### CircMETTL3 is highly expressed in sHLH patient plasma exosomes

**Fig. 1:**
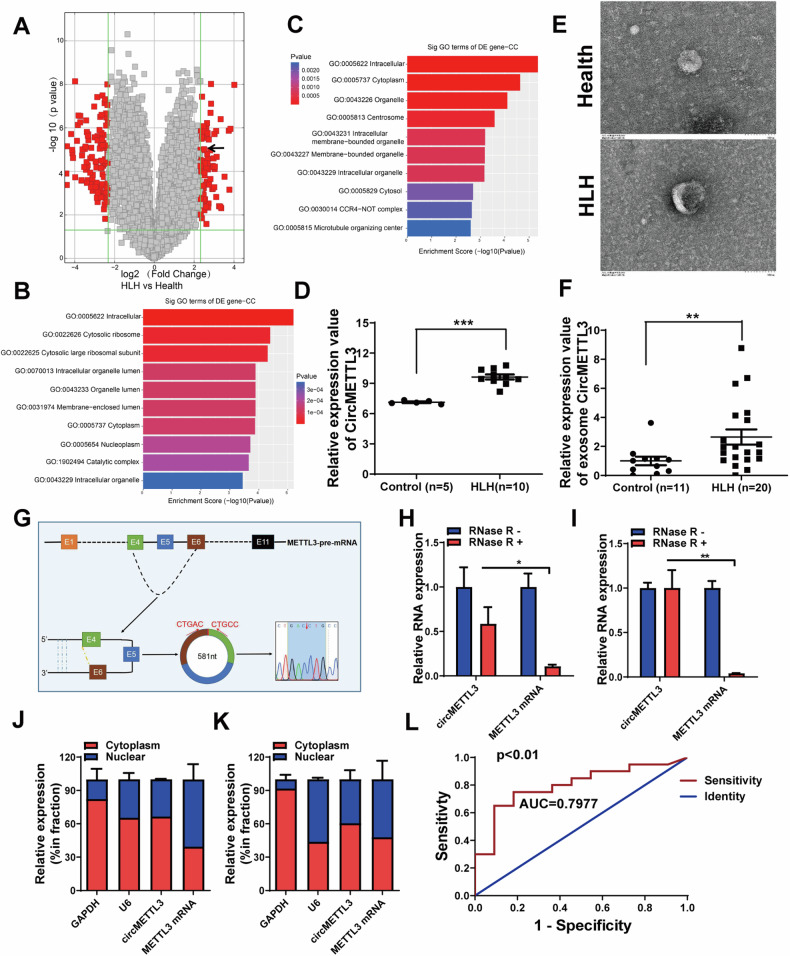
Identification and characteristics of exo-circMETTL3 in sHLH patient plasma. **A** 5 health controls and 10 sHLH patients plasma were subjected to Circular RNA chip analysis. The volcano plot shows the differentially expressed circular RNA in the plasma of children with sHLH (circMETTL3 was marked with a black arrow, *p* < 0.01 and fold change > 5). GO pathway enrichment analysis of up-regulated circular RNA (**B**) and down-regulated circular RNA (**C**). **D** The expression of circMETTL3 in the 5 health controls and 10 sHLH patients plasma by Circular RNA chip quantification. Data have been represented as mean ± standard deviation (SD). ***p < 0.001. **E** Identification of characteristics of of healthy and sHLH plasma exosomes by electron microscopy (Scale bar = 100 nm). **F** The expression of exo-circMETTL3 in the 11 health controls and 20 sHLH patients plasma by qRT-PCR. Data have been represented as mean ± SD. *p < 0.05. **G** The schematic diagram of the formation of circMETTL3 through reverse splicing of exons 4-6 of pre- mRNA of METTL3. **H**, **I** Evaluate the level of linear METTL3 or circMETTL3 after RNase R treatment by qRT-PCR. Data have been represented as mean ± SD. *p < 0.05, **p < 0.01. **J**, **K** Analyzing the cytoplasmic and nuclear distribution of CircMETTL3 through cell fractionation in 293 T and THP-1 cells. GAPDH or U6 were used as cytoplasmic or nuclear markers, respectively. **L** Receiver operating curve (ROC) curve analysis of plasma exo-circMETTL3 as a diagnostic biomarker for sHLH sensitivity analysis.

To screen differentially expressed circRNAs in sHLH patient plasma exosomes, circRNA microarray sequencing of plasma samples from 5 healthy controls and 10 sHLH patients showed that 80 circRNAs were upregulated and 112 circRNAs were downregulated in sHLH patients relative to healthy controls (Figs. 1A and S1A–B). To investigate the potential molecular functions of these circular RNAs, we performed a GO functional enrichment analysis and found that the differentially expressed circular RNAs were associated with membrane-bound organelles (Fig. 1B, C). Among these circRNAs, we found that circMETTL3 was significantly upregulated in the plasma of sHLH patients (Fig. 1D). Exosomal membranes are capable of directly fusing with cell membranes to transmit information [23], suggesting that cirMETTL3 may use exosomal transport to participate in intercellular signaling during the pathogenesis of sHLH. Therefore, TEM (Fig. 1E), NTA (Fig. S1C), and western blotting (Fig. S1D) were used to characterize the circulating plasma exosomes. The plasma exosome concentrations were higher in sHLH patients than in healthy controls. Then, we further obtained plasma exosomes from 20 sHLH patients and 11 healthy children and observed that circMETTL3 was significantly upregulated in the plasma exosomes from sHLH patients by RT‒qPCR (Fig. 1F). The qPCR products were sequenced by Sanger sequencing, and the circMETTL3 expressed in the plasma exosomes of sHLH patients was tentatively determined to be composed of exons 4 and 6 of the pre-METTL3 RNA transcribed from the METTL3 gene, which were spliced end-to-end (Fig. 1G). As verified by in vitro cell experiments, the expression level of circMETTL3 was relatively stable in RNase R-treated cells for 24 hours, while the level of METTL3 mRNA was markedly decreased (Fig. 1H, I). Cytoplasmic nucleus isolation experiments revealed that circMETTL3 was distributed in both the cytoplasm and nucleus, with a predominance in the cytoplasm (Fig. 1J, K). Intriguingly, the ROC curve assessed circMETTL3 as a diagnostic biomarker for sHLH with an area under the curve (AUC) value of 0.7977 (Fig. 1F).

### Exosomes carrying circMETTL3 promote the polarization and activity of THP-1 cells in vitro

**Fig. 2:**
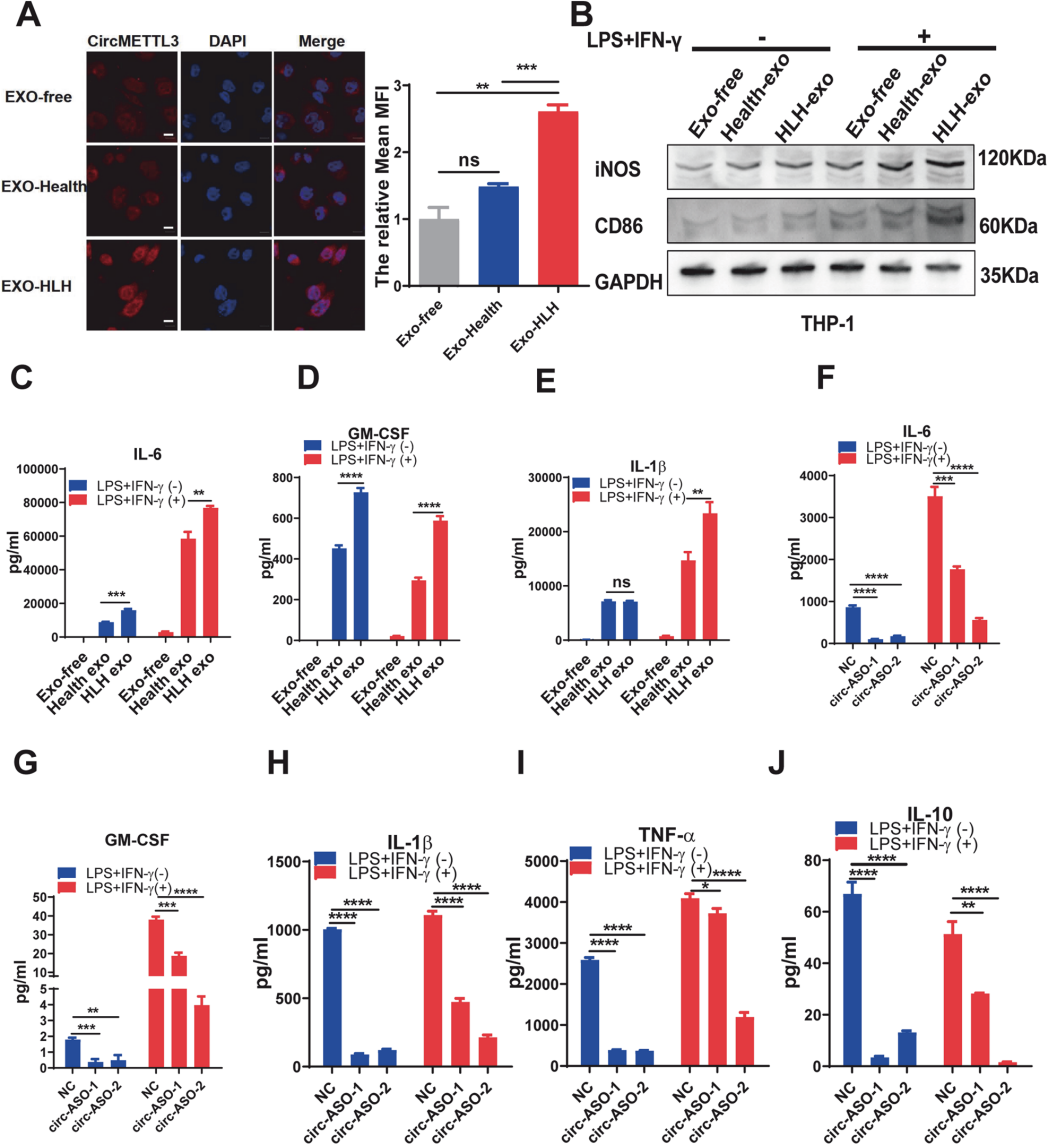
Exosome-circMETTL3 promotes macrophage M1 polarization. **A** The level of circMETTL3 was detected using the Fish assay in THP-1 cells pretreated with PMA and subsequently added with exosomes from health and sHLH pediatric plasma for 24 hours. Error bars represent mean ± SD from *n* = 3 independent experiments. **B** Western blotting was used to examine the expression of iNOS and CD86 in THP-1 cells pretreated with PMA after stimulation with the addition of healthy and HLH exosomes for 6 hours and then treated with or without LPS for 24 hours. **C**–**E** Multi-Cytokine Kit Analysis the cytokines level (IL-6, GM-CSF, IL-1β) of PMA-differentiated THP-1 cells after health and HLH plasm exosome stimulation with or without LPS/IFN-γ treatment. **F**–**J** The cytokines level (IL-6, GM-CSF, IL-1β,TNF-ɑ, IL-10) of PMA- differentiated THP-1 cells after knock down circMETTL3 with or without LPS/IFN-γ treatment. (In **C**–**J**, error bars represent mean ± SD from *n* = 3 independent experiments, *p < 0.05, **p < 0.01, ***p < 0.001, ****p < 0.0001, n.s. not significant).

Macrophage hyperactivation is a critical factor contributing to the immune system disorder of HLH [24]. Firstly, we analyzed the single-cell data of macrophage proportion in peripheral blood mononuclear cells (PBMC) of patients with HLH, and the results showed that the proportion of macrophage in HLH patients was higher than that in the healthy control group (Fig. S2A–C). Moreover, our flow cytometry results suggested a high proportion of M1 phenotype of macrophages in the peripheral blood of children with sHLH (Fig. S2D). These findings suggest that aberrant proliferation and activation of macrophages is present in sHLH. To further clarify the link between sHLH macrophage activation and plasma exosome circMETTL3 expression, we stimulated THP-1 cells with plasma exosome samples from healthy children and sHLH patients for 48 hours. Then, a significant increase in circMETT3 levels in THP-1 cells was observed by RNA-FISH assay after stimulation of THP-1 cells by sHLH plasma exosomes (Fig. 2A). This result indicates that sHLH plasma exosomes upregulated the expression of circMETTL3 in macrophages. LPS combined with IFN-γ is a classical agent for inducing M1 polarization in macrophages [25]. Under the synergistic effect of LPS and IFN-γ, sHLH plasma exosomes promotes the M1 macrophage shift, which was verified by the protein levels of iNOS and CD86 (Fig. 2B). With the activation of macrophages, large amounts of inflammatory factors were released, including IL-6, GM-CSF, IL-1β (Fig. 2C–E). These results indicate that upregulated circMETTL3 in the plasma exosomes of children with sHLH directly promotes the M1 polarization of macrophages and enhances the activation of macrophages induced by LPS and INF-γ. To further investigate the biological function of circMETTL3 in THP-1 cells, small interfering RNAs (METTL3-siRNAs and METTL3-ASOs) specifically targeting the back-splice region of circMETTL3 were designed. Subsequently, qRT‒PCR showed that among the small interfering RNAs we designed, circMETTL3-siRNA and circMETTL3-ASO effectively decreased the level of circMETTL3 in THP-1 cells, but not the protein expression of full length METTL3 (Fig. S3A, B). Whereas, they did not affect the mRNA level of intracellular TNF-ɑ with LPS and IFN-γ costimulation (Fig. S3C). However, analysis of the cell supernatant showed that the levels of cytokines secreted by the cells were significantly decreased (Fig. 2F–J). Next, after overexpression of circMETTL3 in 293 T and THP-1 cells, cell supernatants were collected to obtain exosomes to stimulate THP-1 cells, and secretion of the cytokines IL-6, GM-CSF, L-1β, and TNF-ɑ were elevated (Fig. S4A, B). Collectively, these results suggest that exosomal circMETTL3 plays an important role in the activation of M1 macrophages.

### Targeting circMETTL3 improves sHLH inflammation by inhibiting the activation of macrophages in vivo

**Fig. 3:**
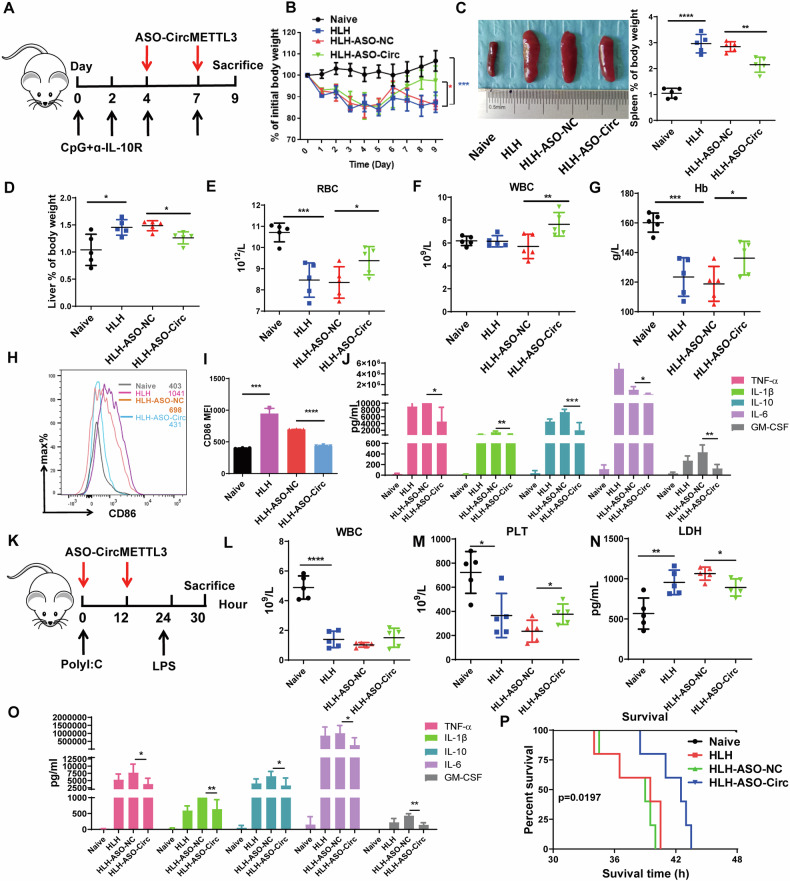
ASO-circMETTL3 treatment ameliorated hypercytokinemia and blood metrics associated with sHLH. **A** Treatment schedules of different mouse groups including saline, ASO-NC and ASO-CircMETTL3 for IL-10R antibody and CPG-induced sHLH mouse models. **B** The change of body weight from all groups for IL-10R antibody and CPG-induced sHLH mouse models. **C**, **D** Image of spleen, spleen% of body weight and Liver% of body weight from all the groups. **E**–**G** The effect of ASO-circMETTL3 on sHLH were investigated by blood routine test indicated by RBC (**E**), WBC (**F**), Hb (**G**) (*n* = 5). Representative data from one experiment out of three independent experiments. *n* = 5, each symbol represents one biological replicates. Data have been represented as mean ± SD. *p < 0.05, **p < 0.01, ***p < 0.001,****p < 0.0001 (**B**–**G**). **H**. FACS analysis of the percentage CD45 + /CD3,CD19-/CD11b + /CD86+ macrophages in blood from different treatment groups. **I**. Analysis of the average fluorescence intensity of CD86. Error bars are mean ± SD for *n* = 3 biological replicates.***p < 0.001,****p < 0.0001. **J**. The effect of ASO-CircMETTL3 on cytokine storm were assessed by quantification of serum levels of multiple cytokines including IFN-γ, TNF-α, IL-12p70, IL-1β, CXCL10, IL-10, IL-6 and GMCSF (*n* = 5). Representative data from one experiment out of two independent experiments. *n* = 5, each symbol represents one biological replicates. Data have been represented as mean ± SD.*p < 0.05, **p < 0.01, ***p < 0.001. **K** Treatment schedules of different mouse groups including saline, ASO-NC and ASO-CircMETTL3 for poly (I:C) and LPS-induced lethal sHLH model. **L**–**N** The effect of ASO-circMETTL3 on sHLH were investigated by blood routine test indicated by WBC (**L**), PLT (**M**) and lactate dehydrogenase (LDH) (**N**) (*n* = 5). **O**. Flow cytometry was used to semi-quantitatively evaluate the levels of cytokines including IFN-γ, TNF-α, IL-12p70, IL-1β, CXCL10, IL-10, IL-6, and GM-CSF in all the groups serum (*n* = 5). Representative data from one experiment out of two independent experiments. *n* = 5, each symbol represents one individual mouse. Data have been represented as mean ± SD, *p < 0.05, **p < 0.01 and ****p < 0.0001 (**L**–**O**). **P** Survival curves of poly (I:C) and LPS-induced sHLH mouse model treated with saline (Naive group) or ASO-NC (HLH-ASO-NC group) or ASO-CircMETTL3 (HLH-ASO-Circ group), (*n* = 5, each symbol represents one individual mouse from one independent experiment. HLH-ASO-NC group was significantly different compared to HLH-ASO-Circ group, *p* = 0.0197).

To further confirm the in vitro findings, we then verified the biological role of circMETTL3 in sHLH in vivo. IL-10R antibody and CPG costimulation can mimic the sHLH inflammatory factor storm to construct an sHLH model. We treated sHLH model mice with a tail vein injection of ASO-circMETTL3 (Fig. 3A). The silencing of circMETTL3 dramatically increased the body weight of mice (Fig. 3B). The spleen/body weight ratio (Fig. 3C) and liver/body weight ratio (Fig. 3D) were significantly improved, as were the blood RBC, WBC and hemoglobin levels (Fig. 3E–G). Importantly, through flow cytometry, the percentage of CD11b + /CD86+ macrophages in the sHLH group increased, while the percentage decreased after ASO targeted circMETTL3 (Fig. 3H, I). Consistent with this finding, the levels of cytokines, including TNF-α, IL-1β, IL-10, IL-16 and GM-CSF, were significantly increased in sHLH model mice but were decreased in ASO-circMETLL3-treated mice (Fig. 3J). Next, we monitored immunohistochemical and immunofluorescence staining of the spleen and liver of the sHLH and sHLH-ASO-NC groups, which revealed increased inflammation in the portal vein and lobules of the liver and increased hemophagocytes and hemorrhagic foci in the spleen (Fig. S5A). Correspondingly, the proportion of F4/80 + /iNOS+ M1 macrophages was significantly enhanced. In contrast, the proportion of F4/80 + /iNOS+ M1 macrophages in the ASO group was remarkably decreased (Fig. S5B). LPS challenge after poly I:C priming can mimic certain aspects of sHLH, a severe systemic inflammatory syndrome that has life-threatening symptoms [26] (Fig. 3K). Consistent with the CPG/IL-10R antibody-induced sHLH model, we observed that the numbers of WBCs (Fig. 3L), PLTs (Fig. 3M), the level of LDH (Fig. 3N) and cytokines (Fig. 3O) were improved in the HLH-ASO- circMETTL3 group compared to the HLH- ASO-NC group after LPS challenge in poly I:C-primed animals. Furthermore, the overall survival of HLH-ASO-circMETTL3-treated mice was prolonged compared to that of HLH-ASO-NC group mice (*n* = 5, mean survival time = 42.5 hours vs. 39.0 hours) (Fig. 3P). Altogether, these data demonstrate that ASO-circMETTL3 may be beneficial treatment that alleviates most of the symptoms of sHLH.

### CircMETTL3 activates macrophages by encoding a 156-amino acid (aa) novel protein, METTL3-156aa

**Fig. 4:**
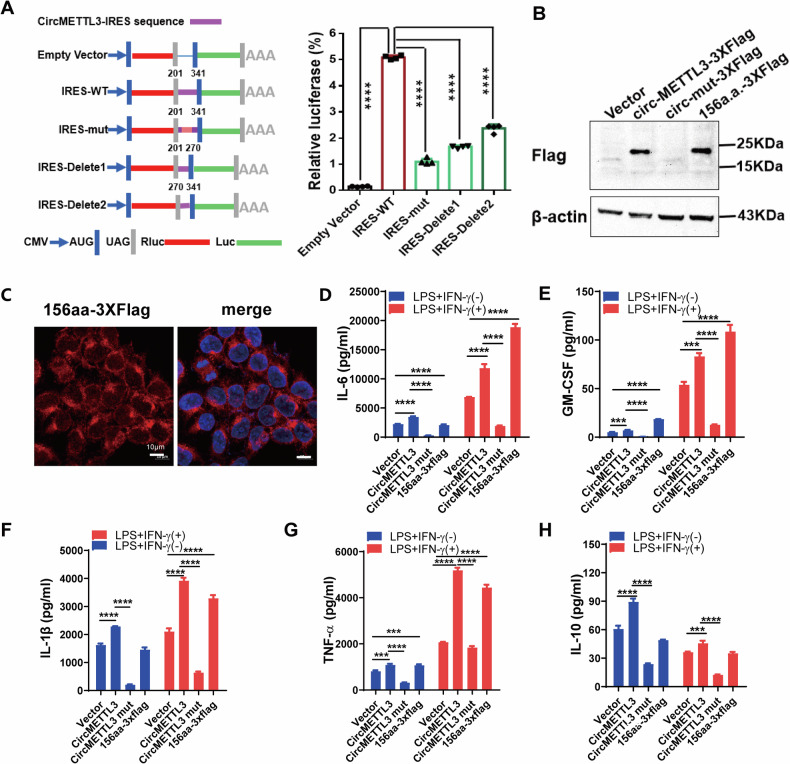
Verification of the coding ability of circMETTL3. **A** Left panel, the wild or mutate type of IRES was cloned between the Rluc and Luc reporter genes with independent start (AUG) and stop (UGA) codons. Right panel, the relative luciferase activity was tested. Data represent the mean ± SD (*n* = 4 independent experiments, ****p < 0.0001). **B** Western blot of 293 T cells with Empty Vector, circ-METTL3-3XFlag vector, splicing donor site mutant vector (circ-mut-3XFlag), and linear-METTL3-156aa-3XFlag vector using Flag antibody. **C** Representative images of cells 293 T with linear-METTL3-156aa-3XFlag vector and stained with anti-Flag antibody (Scale bar=10 μm). All experiments were repeated at least three times. **D**–**H** Expression levels of cytokines IL-6, GM-CSF, IL-1β, TNF-α and IL-10, after THP-1 cells are transfected with different circMETTL3 vectors. Error bars represent mean ± SD from *n* = 3 independent experiments, ***p < 0.001,****p < 0.0001.

To further explore the potential functional mechanism of circMETTL3 in macrophage activation, we found no enrichment of circMETTL3 through RNA immunoprecipitation (RIP) experiments involving AGO2, which mediates interactions between target RNA and miRNA [27] (Fig. S6A). According to the prediction results of the online database circRNADb, an open reading frame (ORF) with the initiation codon ATG and an IRES at 274-327 nt were contained in the sequence of circMETTL3, which encodes a protein of 156 amino acids (Fig. S6B). Furthermore, through sucrose gradient ultracentrifugation to obtain RNA associated with polysome, followed by qPCR analysis. We found that the exosomal circMETTL3 and HLH exosomes increased the cirMETTL3 level in translation active polysomes, further suggesting that exosomal circMETTL3 has a function in protein translation (Fig. S6C–D). This observation suggests that circMETTL3 has the potential to encode a protein, which was termed METTL3-156aa in this study. To verify the activity of the predicted IRES in circMETTL3, a dual-luciferase assay was performed, which showed that the luciferase activity of the wild-type IRES reporter was significantly increased compared with that of the mutated IRES reporter (Fig. 4A). Moreover, the METTL3-156aa product shares the same sequence at aa 284–435 with wild-type METTL3, but it contains the special C-terminal sequence SVRRS. To further confirm the existence of METTL3-156aa, we transferred the the Flag-labeled circMETTL3 overexpression plasmid and the Flag-labeled liner-METTL3-156aa plasmid into HEK293T cells (Fig. 4B). The silver staining results showed that there was an obvious protein band at a molecular weight of 20 kDa, which was exactly the predicted size of METTL3-156aa (Fig. S7A). The 20 kDa protein band was excised and subjected to LC‒MS/MS. MS detected the sequence of KLHFRR, which was present in METTL3-56aa (Fig. S7B). An immunofluorescence assay using an anti-Flag antibody confirmed that METTL3-156aa-Flag was located in the cytoplasm of THP-1 cells overexpressing the METTL3-156aa plasmid, as shown in Fig. 4C. The levels of the THP-1 cell supernatant cytokines IL-6, GM-CSF, IL-1β, TNF-α and IL-10 were enhanced after transfection with the overcircMETTL3 and METTL3-156aa vectors but decreased in the mut-circMETTL3 vector group (Fig. 4D–H). Overall, circMETTL3 could activate macrophages by encoding a novel protein, METTL3-156aa.

### METTL3-156aa promotes M1 polarization and aggravates the features of sHLH

**Fig. 5:**
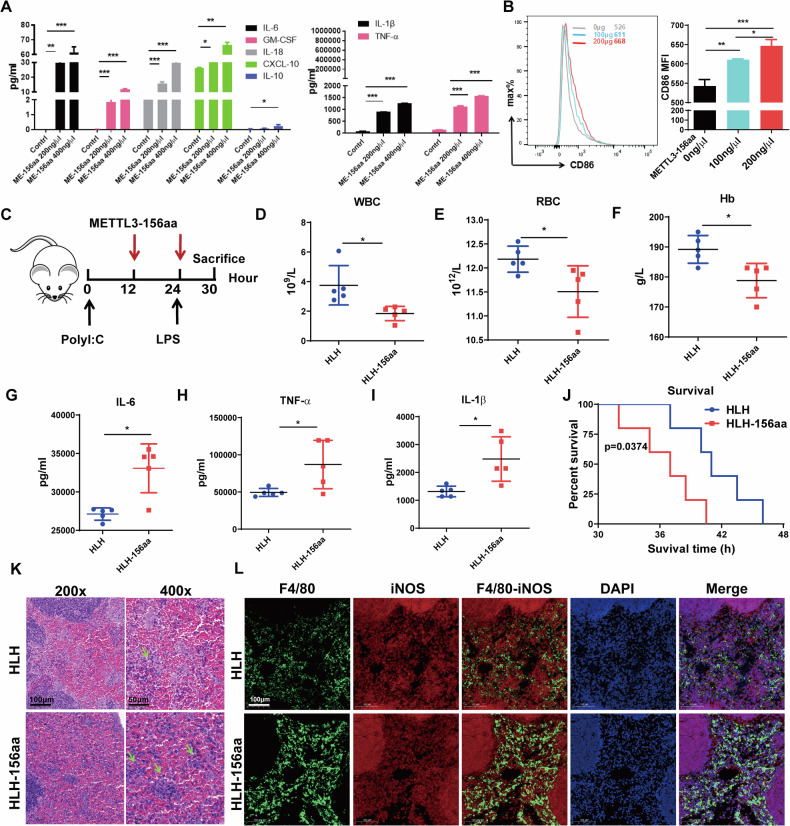
Polypeptide METTL3-156aa aggravates sHLH in a lethal model. **A** The effect of METTL3-156aa on sHLH cytokine storm were assessed by quantification of cell supernatant levels of multiple cytokines including IL-6 and GM-CSF, IL-18, CXCL10, IL-10, IL-1β, TNF-α. Data represent the mean ± SD (*n* = 3 independent experiments, *p < 0.05, **p < 0.01, ***p < 0.001). **B** FACS analysis of the percentage CD11b + /CD86+ macrophages in THP-1cells with or without METTL3-156aa treatment. Error bars represent mean ± SD from *n* = 3 independent experiments, *p < 0.05, **p < 0.01, ***p < 0.001. **C** Treatment schedules of different groups including saline and METTL3-156aa for poly (I:C) and LPS-induced lethal sHLH model. **D**–**F** The effect of METTL3-156aa on sHLH were investigated by blood routine test indicated by WBC (**D**), RBC (**E**), Hb (**F**) (*n* = 5). **G**–**I** The effect of METTL3-156aa on IL-6 (**G**), TNF-α (**H**) and IL-1β (**I**) level were used to semi-quantitatively evaluate by flow cytometry (*n* = 5). Representative data from one experiment out of two independent experiments. *n* = 5, each symbol represents one individual mouse. Data have been represented as mean ± SD, *p < 0.05 (**D**–**I**). **J** Survival curves of sHLH mouse model treated with saline or METTL3-156aa. *n* = 5, each symbol represents one individual mouse from one independent experiment. HLH group was significantly different compared to HLH-156aa group, *p* = 0.0374. **K** Splenics were stained with H&E in HLH and HLH-156aa group (Green arrow, inflammation. Scale bar: 200×,100 μm; 400×, 50 μm). **L** Splenic M1 polarization of macrophages were stained by immunofluorescence for macrophage (F4/80) and M1 macrophage (iNOS) markers in HLH and HLH-156aa group (Scale bar, 100 μm).

To further investigate the biological function of METTL3-156aa, we added the polypeptide METTL3-156aa to PMA-induced THP-1 cells in vitro. As expected, when THP-1 cells were treated with METTL3-156aa, the levels of cytokines, including IL-6, GM-CSF, IL-18, CXCL10, IL-10, IL-1β, and TNF-α (Fig. 5A), and the percentage of CD11b + /CD86+ macrophages were dramatically increased (Fig. 5B). Moreover, by intraperitoneal injection of poly I:C and LPS to construct an lethal sHLH model and then injecting METTL3-156aa into the tail vein, we found a further decrease in WBCs, RBCs, and Hb trilineage levels (Fig. 5D–F). Furthermore, IL-6 (Fig. 5G), TNF-α (Fig. 5H) and IL-1β (Fig. 5I) levels increased in the HLH-156aa group. Subsequently, we found that compared to the HLH group, the HLH-156aa group had increased inflammation in the spleen by immunohistochemistry (Fig. 5K). Immunofluorescence staining showed a significant increase in F4/80^+^/iNOS^+^ cells stimulated with METTL3-156aa (Fig. 5L). Furthermore, the overall survival of the METTL3-156aa-treated HLH group was shorter than that of the HLH group (*n* = 5, mean survival time = 36.5 hours vs. 41.5 hours) (Fig. 5J). In summary, the effects of circMETTL3 on the proinflammatory biological function of macrophages depend on its encoded protein METTL3-156aa.

### METTL3-156aa interacts with LDHA and remodels macrophage glycolysis to induce M1 polarization

**Fig. 6:**
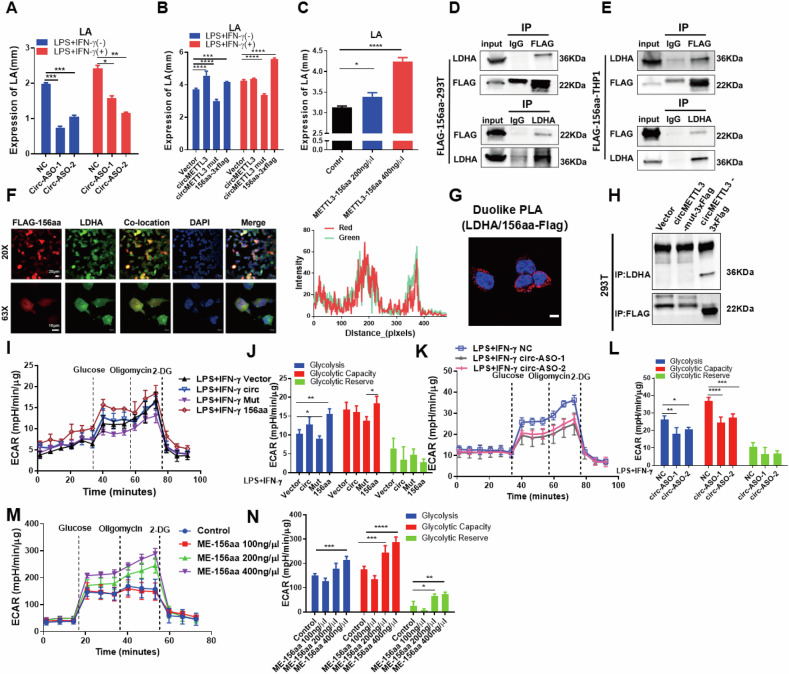
METTL3-156aa promotes macrophage glycolysis by binding to LDHA. **A**–**C** Lactate levels of THP1 cell supernatants after knockdown of circMETTL3 (**A**), overexpression circMETTL3 (**B**) or addition of METTL3-156aa (**C**). Data are shown as mean ± SD (*n* = 3 independent experiments, *p < 0.05, **p < 0.01, ***p < 0.001, ****p < 0.0001). **D**, **E** Mutual interaction of LDHA and Flag-METTL3–156aa in HEK293T (**D**) and THP-1 (**E**) cells were determined by IP. **F** Flag-tagged METTL3–156aa was transfected into HEK293T cells and immunofluorescence was performed using anti-Flag and anti-LDHA antibody. Scale bar: 20x, 20 μm; 63x, 10 μm. All experiments were repeated at least three times. **G** The in situ PLA was performed to examine the interaction between LDHA and METTL3-156aa (scale bar, 10 μm). All experiments were repeated at least three times. **H** Enhanced levels of METTL3-156aa binding to LDHA after transfection of circMETTL3 vector in HEK293T cell line. **I**–**N** The ECAR was determined using a Seahorse XF96 analyzer to evaluate glycolytic flux after overexpression circMETTL3, knockdown of circmettl3, or addition of METTL3-156aa. Glycolysis, glycolytic capacity, and glycolytic reserve were determined by the sequential addition of 10 mM glucose, 1 mM oligomycin, and 50 mM 2-D-glucose. Values represent the mean ± SD of at least three independent experiments (*p < 0.05, **p < 0.01, ***p < 0.001).

To investigate the molecular mechanism by which METTL3-156aa promotes M1 macrophage polarization, we transfected the Flag-labeled METTL3-156aa plasmid into HEK293T cells and THP-1 cells. The potential interacting protein was pulled down in the Flag-tagged METTL3-156aa immunoprecipitation complex (Fig. S7C). Protein MS analysis was used to identify the differentially expressed proteins, and they were found to be significantly enriched in the glycolytic metabolic pathway by KEGG analysis (Fig. S7D). Similarly, the level of glycolysis metabolite lactate was decreased after knocking down circMETTL3. However, it was increased after overexpression with the circMETTL3 and METTL3-156aa vectors (Fig. 6A, B). In addtion, lactate was rised with the addtion of METTL3-156aa (Fig. 6C). These suggest that circMETTL3/METTL3156aa may affect glycolysis metabolism. Importantly, in the list of ranked proteins that were identified to be related to glycolysis, the protein with the highest abundance was LDHA, revealing that LDHA was a potential interacting protein of METTL3-156aa (Fig. S7E). Furthermore, Flag-tagged METTL3-156aa could mutually interact with LDHA, confirming the direct interaction between METTL3-156aa and LDHA by Co-IP (Fig. 6D, E). Subsequent immunofluorescence staining further showed colocalization between FLAG-156aa and LDHA. (Fig. 6F). Similarly, the proximity ligation assay further suggests that these two proteins interact closely in the cytoplasm (Fig. 6G). Additionally, we overexpressed circMETTL3 with or without the IRES mutation in 293 T cells, and Co-IP experiments revealed that the interaction between the encoded peptide METTL3-156aa and LDHA was not observed in the IRES mut group (Fig. 6H). LDHA is a glycolytic rate-limiting enzyme that mediates the production of lactate from pyruvate. We overexpression circMETTL3 and found that the enzyme activity of LDH was enchanced (Fig. S8A). Moreover, overexpression of circMETTL3 was found to enhance the glycolytic level of THP-1 cells after PMA induction under LPS and IFN-γ costimulation (Fig. 6I, J). However, ASO-specific knockdown of circMETTL3 decreased glycolysis, glycolysis capacity and glycolysis reserves (Fig. 6K, L). The addition of the METTL3-156aa peptide directly reversed glycolytic flux (Fig. 6M, N). Thus, the above outcomes showed that METTL3-156aa binds to LDHA and that lactate levels are increased, remodeling macrophage glycolysis. To further elucidate that the METTL3-156aa mediating the activation of glycolysis is derived from exosomal-circMETTL3. We used exsomal circMETTL3 to treat THP-1 cells. The western blot revealed an unsignificant alterations in the protein levels of LDHA and PKM2, suggesting that METTL3-156aa plays a role in regulating the enzyme activity of LDHA to promote glycolysis (Fig. S8B). Subsequently, we treated THP1 cells with exosomes released from 293 T cells with circMETTL3 or METTL3 knockdown. We found that the glycolytic flux of THP-1 cells decreased after interfering with circMETTL3. However, knocking down METTL3 did not show a significant change in glycolytic flux (Fig. S9C, D). Importantly, protein analysis demonstrated an increase in the levels of M1-activating protein iNOS/CD86 with the addition of the peptide METTL3-156aa and a decrease in the levels of iNOS/CD86 levels with the further addition of the glycolysis inhibitor 2-DG (Fig. S9E). The cytokines production was not restore in THP-1 cells treated with exosomal circMETTL3 in the presence of shRNA LDHA (Fig. S9A–F). These findings suggest that the exosomal circMETTL3-encoded METTL3-156aa reshapes glycolytic metabolism and activates M1 macrophages by increasing LDHA enzyme activity.

Next, we further explored the binding sites of LDHA with peptides. The METTL3-156aa binding site to LDHA by database modeling and found that the binding site was not at the specific amino acid sequence of METTL3-156aa (SVRRS) but at an amino acid site shared with the protein METTL3 encoded by the parent gene METTL3 (Fig. S10A). Immediately after this analysis, we demonstrated by Co-IP that LDHA did not bind to METTL3 (Fig. S10B). Additionally, the increase of lactate is stronger in METTL3-156aa overexpressing samples than the overexpression of SVRRS site-mutated METTL3-156aa and METTL3 plasmid, further demonstrating that LDHA binds to the polypeptide METTL3-156aa and that the five amino acids of SVRRS were disordered sequences. Whether they bind to LDHA by phase separation needs to be further investigated (Fig. S10C).

### Glycolytic metabolite lactate-induced lactylation modifies SRSF10 to promote circMETTL3 ring formation

**Fig. 7:**
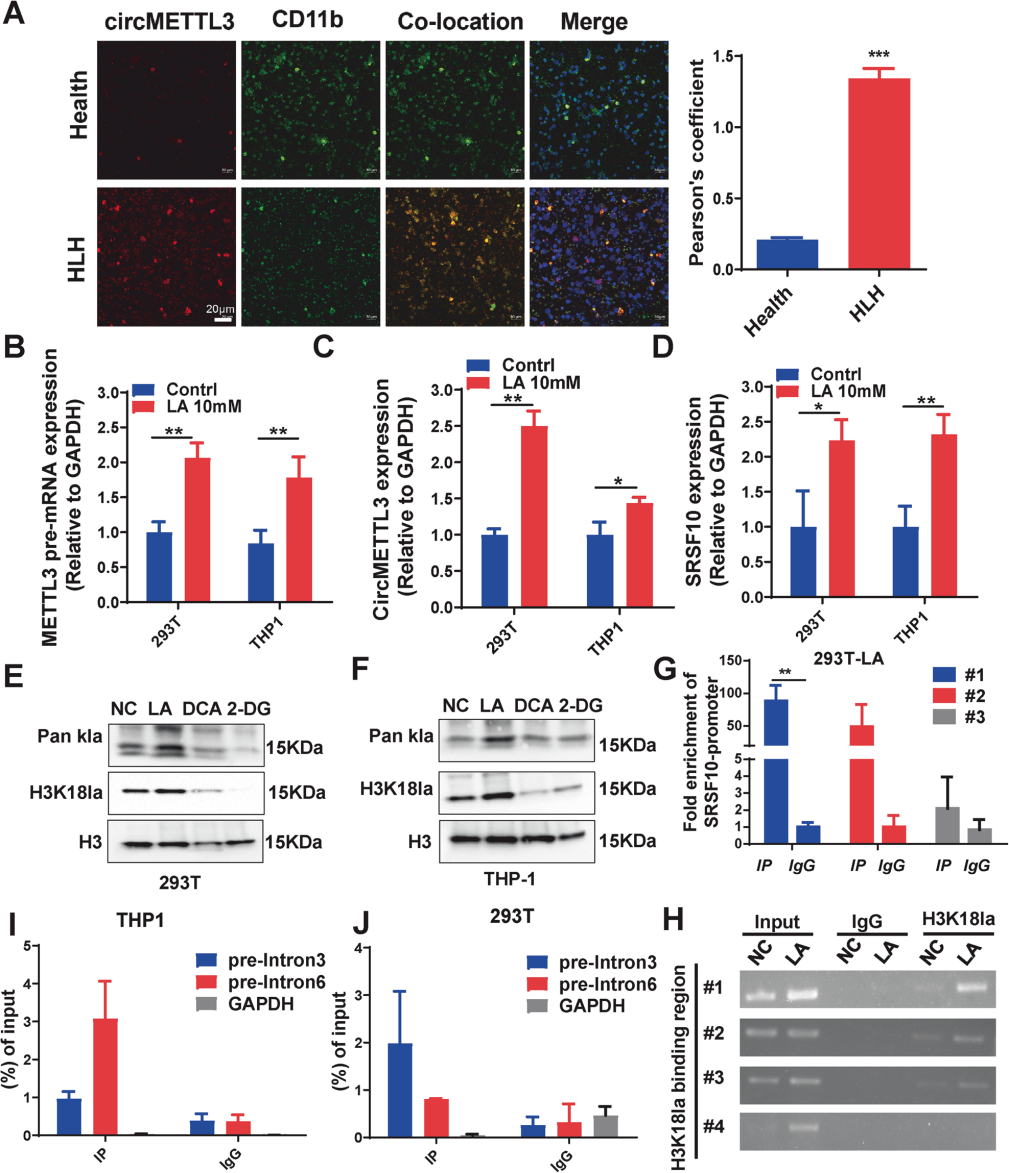
SRSF10 upregulates the expression of *circMET*TL3 by lactylation modification. **A** The expression of circMETTL3 in CD11b+ monocytes from health and HLH blood anlysised by immunofluorescence (scale bar, 20μm). Error bars are mean ± SD for *n* = 3 biological replicates, ***p < 0.001. **B** The expression of *METTL3* pre-mRNA was examined by qRT-PCR in HEK293T and THP-1 cells with or without lactate treatment. **C** The expression of *circMETTL3* was examined by RT-qPCR in HEK293T and THP-1 cells with or without lactate treatment. **D** The expression of *SRSF10* was examined by qRT-PCR in HEK293T and THP-1 cells with or without lactate treatment. Error bars represent mean ± SD from *n* = 3 independent experiments, *p < 0.05, **p < 0.01 (**B**–**D**). **E**, **F** Western blot was used to determine the lactation levels HEK293T and THP-1 cells after treatment with Lactate, Sodium dichloroacetate (DCA) and 2-Deoxy-D-glucose (2-DG). **G**, **H** ChIP-qPCR and ChIP-PCR analysis the H3K18la was enriched in the *SRSF10* promoter region. **I**, **J** SRSF10 bound to the fanking intron sequence of *circMETTL3* detected by RIP assay. Intron 3 and intron 6 were selected for RIP experiments using the SRSF10 antibody and referred to the SRSF10 binding site of *METTL3* pre-mRNA in THP-1 and 293T cells. Data represent mean ± SD of triplicate independent experiments, **p < 0.01 (**G**, **I**, **J**).

To further trace the source of high circMETTL3 expression in HLH, we found a significant increase in circMETTL3 expression levels in CD11b-labeled mononuclear macrophages from sHLH patients relative to healthy controls by fluorescence colocalization (Fig. 7A). Then, lactate was found to positively regulate the expression of METTL3 pre-mRNA and circMETTL3 in THP-1 cells (Fig. 7B, C). The above results suggest that lactate may regulate circMETTL3 formation via a positive feedback mechanism. Commonly, circRNA is produced by the alternative splicing of pre-mRNA [28]. Splicing factors act on the RNA surrounding the circRNA sequence on the pre-mRNA strand to initiate splicing and upregulate the expression of circRNA [29]. Here, we found that lactate could also upregulate the mRNA expression of SRSF10 (Fig. 7D). Similarily, the western blot results showed a significant increase in the protein level of SRSF10, as well as METTL3-156aa, after the addition of 10 mM lactic acid (Fig. S11A). Subsequently, we investigate the mechanism of by which lactate upregulates SFSR10. However, knocking down SRSF10 and then adding 10 mM lactic acid to stimulate the cells did not increase the level of circMETTL3, indicating that lactic acid increases the level of circMETTL3 by regulating SRSF10 (Fig. S11B). Further explore the mechanism by which lactate increases SRSF10 levels. We found that the addition of lactic acid increased the level of the H3K18la protein at the histone modification site and that the glycolysis inhibitors DCA and 2DG could inhibit this lactylation (Fig. 7E, F). ChIP‒qPCR analysis also showed that H3K18la levels were enriched in the *SRSF10* promoter region, suggesting that *SRSF10* transcription may be activated by H3K18la (Fig. 7G, H). Then, we used the RBPmap database to predict the optimal sequences of METTL3 pre-mRNA introns 3 to 6 that might bind to SRSF10 (*RBPmap data:* http://rbpmap.technion.ac.il/). The RIP assay confirmed the binding of SRSF10 to the flanking introns of circMETTL3 (Fig. 7I, J). Altogether, these results indicate that lactate is involved in the formation of circMETTL3 by modulating SRSF10 expression. When SRSF10 was knocked down in THP1 and 293 T cells, the expression of METTL3-156aa was downregulated (Fig. S12A, B). The positive feedback of the circMETTL3/METTL3-156aa/LDHA/glycolysis/ M1 macrophage activation/lactate axis may play crucial roles in HLH development.

## Discussion

HLH has a complicated etiology, and there is a rapid onset and progression of the disease, making it particularly challenging to diagnose and assess individual medications. The 30-day mortality rate is still as high as 14%-30% [30], and the 5-year survival rate is 56%-67%, making it a critical illness that seriously threatens the lives of children [5]. Secondary HLH is a fatal complication related to infections with unknown etiology. Research on sHLH immune dysfunction and the mechanisms that induce cytokine storms is sparse. The hyperinflammatory phenotype of sHLH [31] is manifested by excessive macrophage activation, which is involved in shaping the immune microenvironment and triggering cytokine storms [32]. Therefore, identifying key regulators of macrophage activation is essential for understanding the hyperinflammatory state of sHLH. In the current study, we elucidated the key role of circMETTL3 as a positive regulator of macrophage activation from the perspective of circular RNA involvement in macrophage glucose metabolism. And emphasized the role of lactate as a metabolite in the progression of sHLH. Consequently, targeting circMETTL3 may be a promising treatment strategy for treating high inflammatory status in sHLH.

Exosomes are pivotal mediators of intercellular communication and carry substances such as RNA, DNA and proteins [33]. Furthermore, circular RNA is enriched in exosomes and is the basis for intercellular communication [34]. Numerous studies indicate that exosomes carrying circular RNAs play an important role in the development and progression of various diseases and can be used as candidate molecules for their diagnosis and treatment [35–38]. In this study, we first demonstrated that circMETTL3, which is derived from METTL3, is upregulated in sHLH patient plasma exosomes and can be offered crucial foundation for the diagnosis of sHLH. CircRNAs modulate the differentiation and function of immune cells and transmit immune signals [39]. CircRasGEF1B regulates macrophage activation by controlling the expression of intercellular adhesion molecule 1 (ICAM-1) during the stimulation of macrophages by LPS, as reported in a previous study [40]. Additionally, circZC3H4 regulates macrophage activation in response to silicon dioxide (SiO_2_) exposure [41]. *Mycobacterium tuberculosis* promotes the action of miR-874-3p on its target gene ATG16L1 by downregulating circTRAPPC6B expression, which suppresses autophagy, thus facilitating its immune escape from macrophages [42]. Collectively, these studies highlight the critical role of circRNAs in regulating macrophage activation and function. Our previous proteomic data showed that the expression of CD14, an important molecule associated with plasma macrophage activation, was upregulated in children with HLH. Therefore, we focused on macrophages and further found that sHLH exosome circMETTL3-activated macrophages exhibit proinflammatory functions and secrete cytokines such as IL-6, TNF-α, IL-18 and IL-1β and the chemokine CXCL10. It is suggested that exosomal circMETTL3 may be an intrinsic mechanism of macrophage M1 polarization to induce an inflammatory factor storm.

Currently, the biological functions and potential molecular mechanisms of circular RNA from METTL3 are unknown. CircRNAs are engaged in transcriptional regulation in the nucleus, efficient microRNA sponging, splicing competition with precursor mRNAs, and circRNA-protein interactions [43, 44]. CircMETTL3 is located on chromosome 14 [45] and has several loop-forming modes, and in this study, we identified for the first time that circMETTL3 is reverse spliced into a loop from exons 4 and 6. Some studies have reported that circMETTL3 has an important role in the progression of breast cancer [45, 46] and colorectal cancer [47] by serving as a “miRNA sponge” and impacting the activity of miRNAs. Here, to initially investigate the downstream regulatory mechanism of circMETTL3, we identified the presence of an ORF and an active IRES sequence of circMETTL3 that encodes a 156 amino acid polypeptide (METTL3-156aa). Further functional experiments verified that mutant IRES, which inhibits METTL3-156aa coding function, caused a reduction in the proportion of M1 macrophages and levels of inflammatory factors, suggesting that circMETTL3 has the potential to encode a peptide that promotes the M1 polarization of macrophages and exerts proinflammatory effects.

Metabolites shape the function and differentiation of immune cells, and metabolic reprogramming plays an important role in meeting the energy requirements of immune cells during the different phases of activation and proliferation [48]. Glucose metabolism not only supports the basic life processes of the body but is also the basis for the immune function of macrophages [49]. In the present study, our results show that circMETTL3 encodes a peptide, METTL3-156aa, that directly interacts with LDHA, a key rate-limiting protein of glycolysis, to drive glycolysis and M1 polarization in macrophages that then exert proinflammatory effects. A recent study of an animal model of poly I:C and LPS coinduced hyperinflammatory sHLH reported findings that are similar to our results, suggesting that hyperinflammatory macrophages are highly dependent on glycolytic metabolism and that treatment with the glycolytic inhibitor 2-deoxyglucose is sufficient to rescue animals with sHLH by significantly suppressing the inflammatory response [50]. Overall, it is proposed that targeting circMETTL3 modulates immunometabolic dysregulation and is an effective treatment strategy to alleviate the sHLH inflammatory response.

The major processes driving loop RNA formation are intron pairing-driven circularization, RNA binding protein (RBP)-driven circularization, and lasso-driven cyclization [51]. SRSF10 is an RBP belonging to the SR protein family and functions as a sequence-dependent splicing regulator that is able to bind to circular RNAs and influence circRNA production [29]. Recent studies have illustrated that c-Myc upregulates the expression of SRSF10, which may facilitate the splicing of CAMSAP1 pre-mRNA to form *circCAMSAP1* [52]. During cellular metabolism, lactate is not only a metabolic by-product, the accumulation of lactate promotes lactation modification of histone lysine residues which may directly promote gene transcription and increase gene expression [53]. In this study, we found that lactate upregulated the expression of SRSF10 by histone lactylation. The high expression of SRSF10 promoted METTL3 pre-mRNA splicing to form *circMETTL3*. It is suggested that circMETTL3 activates macrophage glycolytic metabolism to induce M1 polarization and lactate accumulation, which enables the shearing of pre-mRNA of METTL3 into a loop to generate circMETTL3 by the histone lactylation modification of SRSF10.

**Fig. 8:**
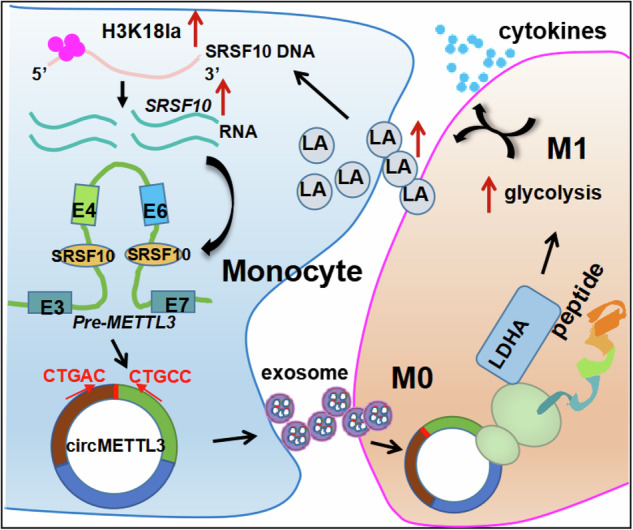
Schematic illustration of *circMETTL3* promoting the M1 polarization of macrophages through positive METTL3-156aa/LDHA/LA/SRSF10 feedback. Exosome-CircMETTL3 encodes the protein METTL3-156aa which binds to LDHA, promoting glycolysis in macrophages. The metabolite lactate increase the level of SRSF10 by lactylation, leading to *METTL3* pre-mRNA transcription and back-splicing under the action of SRSF10, forming a positive feedback on *circMETTL3* production in monocytes, resulting in M1 polarization and cytokines storm.

In conclusion, our study identified a novel peptide METTL3-156aa encoded by circMETTL3 and is an activator of the induction of M1 polarization in macrophages leading to HLH inflammatory factor storm. We revealed the mechanism that METTL3-156aa interacts with LDHA in macrophages to induce glycolysis to M1 polarization in macrophages (Fig. 8). Hence, circMETTL3 can serve as a novel biomarker, helping to elucidate the new mechanism of sHLH pathogenesis from the perspective of circular RNA involvement in immune metabolism. Furthermore, circMETTL3 may be a promising therapeutic target for sHLH.

## Materials and methods

### Clinical samples

Freshly preserved plasma was mainly obtained from children with hemophagocytosis and healthy control children from Hunan Provincial Children’s Hospital from 2018 to 2022 years. All patients have signed informed consent and are approved by the ethics committee (HCHLL-2018).

### Cells culture

Human acute monocytic leukemia cell lines (THP1) cells were cultured with RPMI-1640 medium (Gibco, USA) containing 10% fetal bovine serum, and 293 T cells were cultured in RPMI-DMEM medium containing 10% fetal bovine serum (BI, Israel), and incubated at 37^o^C in a humidified incubator with 5% CO_2_.

### CircRNA microarray

Five pediatric sHLH blood samples and 5 healthy donor blood samples were examined using the circRNA microarray provided by Kangcheng Bio-Tech Inc. The sample preparation and microarray hybridization were performed based on Arraystar’s standard protocols. A circRNA microarray (Arraystar Human circRNAs chip, ArrayStar) containing more than 14000 probes specific for splice sites in human circRNAs was used in this study. R software was used to process the subsequent data after normalization. Differentially expressed circRNAs were identified through volcano plot filtering and fold change filtering. CircRNAs with a fold change ≥5.0 or ≤-5.0 and a *p* value < 0.05 were identified as significantly differentially expressed circRNAs.

### Exosomes and exosome RNA isolation

We used ultracentrifugation with a Beckman ultrahigh-speed centrifuge to obtain exosomes. Plasma samples were rapidly heated at 37 °C, centrifuged at 2000 × *g* and 4 °C for 30 min to remove cellular deposits, and centrifuged at 10,000 × *g* and 4 °C for 45 min to remove larger vesicles. Then, the plasma was filtered through a 0.45 μm filter membrane, and the filtrate was transferred to a new centrifuge tube and centrifuged for 70 min at 100,000 × *g* and 4 °C with the rotor selected for ultrahigh-speed centrifugation. Next, after discarding the supernatant, the samples were centrifuged for 70 min at 4 °C and 100,000 × *g* again after resuspension with 10 mL of precooled 1× PBS. Finally, the supernatant was removed, and exosomes were obtained by resuspension with 100 μL of prechilled 1× phosphate-buffered saline (PBS). Subsequently, the typical pea-like structure of exosomes was observed using transmission electron microscopy (TEM, Hitachi HT-7700), NTA (PARTICLE METRIX, ZetaVIEW) for particle size and exosome concentration analysis, and western blotting with CD81, CD9, and calnexin indicators to identify the purity of exosomes The remaining exosomes were stored at −80 °C.

An Exosome Extraction & RNA Isolation Kit (Rengen Bioscience, EXORNA50-1) was used to extract exosomal RNA from plasma. A two-part method was performed according to the operating instructions to obtain exosomes first and then exosomal RNA was isolated for quantitative real-time polymerase chain reaction (qRT‒PCR) analysis.

### RNA preparation, treatment with RNase R and qRT‒PCR

Total RNA was isolated with TRIzol reagent (Invitrogen) in accordance with the manufacturer’s protocol and cultured with 3 U/mg RNase R (Epicenter Technologies, USA) for 20 min at 37 °C. Five hundred nanograms of RNA was converted to cDNA by reverse transcription with the HiScript III First Strand cDNA Synthesis Kit (Vazyme, Nanjing, China). The cDNA samples were then subjected to real-time quantitative PCR using GoTaq® qPCR Master Mix (Promega, Beijing, China) and optimized for analysis by a PCR instrument (ABI7500, USA). The primers used are listed in Table S1.

### Nuclear and cytoplasmic fractionation

The nuclear and cytoplasmic fractions were extracted using a PARIS^TM^ Kit protein and RNA isolation system (Thermo Fisher Scientific) according to the manufacturer’s protocol [52]. The validity of nuclear and cytoplasmic isolation was evaluated by measuring the protein levels of U6 and GAPDH, which are exclusively displayed in the nucleus and cytoplasm, respectively.

### Western blot analysis

Cells were collected and washed three times with PBS, lysed with immunoprecipitation (IP) lysis buffer (Thermo Scientific, MA, USA) containing a protease inhibitor cocktail for 30 min on ice, and centrifuged at 12,000 × *g* for 15 min at 4 °C. The protein concentration of the supernatant was calculated with a BCA protein assay kit (Pierce, Rockford, IL), and the protein samples were separated using polyacrylamide gel electrophoresis containing SDS and transferred to polyvinylidene fluoride (PVDF) membranes (Millipore, Billerica, MA, USA). Following one hour of blocking with 5% BSA in PBS buffer, the membranes were incubated with the primary antibody overnight at 4 °C. Next, the membrane was placed on a shaker and washed 3 times with TBST buffer for 10 minutes each. Subsequently, the membrane was incubated with the corresponding HRP-labeled secondary antibody for 1 hour at room temperature and washed 3 times with TBST buffer on a shaker. Finally, blot signals were detected by Amersham Imager 600 (General Electric, USA) with Supersignal^TM^ West Atto hypersensitive substrates (Thermo Scientific, A38555).

### RNase R treatment

Experiments were conducted by RNase R (Thermo Fisher, Grand Island, NY, USA) processing to validate the stability of circMETTL3. Total RNA was obtained from 293 T and THP-1 cells and separated into two groups. One group was subjected to incubation with 1 μL of RNase R (20 U/μL; Epicenter, WI, USA) at 37 °C for 30 min, while the other group was processed without RNase R. The RNA was isolated by incubation at 70 °C for 10 min to inactivate RNase R and then inverted for RT‒PCR assay. The primers used are shown in Table S1.

### Flow cytometry

A total of 1×10^6^ cells were collected and washed three times with PBS. Then, each sample was resuspended in a 200 μL PBS mixture containing the following detection antibodies: anti-mouse/human CD11b (BioLegend, USA), anti-mouse F4/80 (BioLegend, USA) and anti-human CD86 (BioLegend, USA). The cells were vortexed and incubated at room temperature for 15 minutes. After antibody staining, cells were collected and cleaned with PBS once. Finally, cells were obtained by resuspension with 200 μL of PBS, and the ratio of macrophage subsets was analyzed by flow cytometry (BD LSRFortessa, USA). For intracellular staining of CD206, after PBS washes, cells were suspended in fixation buffer (BioLegend, USA) for 30 min at 4 °C and then at room temperature for 90 min.

### Plasmids and transfection

The circMETTL3 overexpression plasmid, circMETTL3-3XFlag-NC, circMETTL3-3XFlag-pLC5-ciR plasmid, circMETTL3-3XFlag-△TTG-pLC5-ciR plasmid, and linear-circMETTL3-3XFlag-156aa plasmid were generated by chemical gene synthesis, and the pLC-ciR△backward circular frame and PCDH-CMV-MCS-EF1a-GFP-puro vector were used as plasmid backbones. Among it, circMETTL3 overexpression plasmid is non-native circular-forming plasmid. And the liner-METTL3-156aa plasmid is linearized sequence cloned into an overexpression vector. The plasmids were transfected with Lipofectamine 3000 (Invitrogen, USA) according to the manufacturer’s instructions.

### IRES activity verification and dual luciferase reporter assay

IRES sequences, truncator sequences and synthetic mutant sequences were amplified and cloned and inserted into the RLUS-IRES-FLUS-pcDNA3.1(+) reporter vector. After 48 hours of transfection, the activity of luciferase was determined using a dual luciferase reporter system. For each transfected well, the firefly luciferase activity was normalized to the Renilla luciferase activity. The experiment was repeated three times independently.

### RNA interference (RNAi) and transfection

The siRNAs or antisense oligonucleotides (ASOs) were acquired from RIBO Biological (Guangzhou, China). The target sequences are listed in Table S1. SiRNA transfection was performed using Lipofectamine™ RNAi MAX Transfection Reagent (Invitrogen, USA) in accordance with the protocol.

### Immunofluorescence

293 T cells and THP-1 cells that had been treated with PMA (phorbol 12-myristate 13-acetate) were placed in specialized cell culture slides in 24-well plates and left to culture overnight at 37 °C and 5% CO_2_ using DMEM or RPMI 1640 medium, respectively. The cells underwent two washes with preheated PBS for 5 minutes each, followed by fixation with 4% formaldehyde and permeabilization with 0.25% Triton X-100 for 15 minutes. Finally, the cells were washed again twice with preheated PBS for 5 minutes each. After incubation with 1% BSA for 30 minutes for blocking, the cells were treated with specific primary antibodies and incubated overnight at 4 °C. Each well was then treated with a secondary antibody (H + L) DyLight594 conjugated affinipure-goat anti-mouse IgG (Boster, 1:200). After washing three times, anti-fluorescence quenching mounting medium (including DAPI) was added to each well and the cells were mounted with cover glass (Beyotime Biotechnology, Shanghai, China). Finally, the cells were viewed and images were captured under a ZEISS LSM800 confocal microscope (Carl Zeiss AG, Germany).

### Fluorescence in situ hybridization (FISH) assays

Well-fixed and permeabilized cells (as above for immunofluorescence) were incubated for 3 hours at 37 °C with the FISH assay prehybridization solution (Boster, China). Next, the cells were hybridized with 8 μM digoxin-labeled circMETTL3 probe (RIBO Biology, China) for 16 h at 37 °C. After washing the cells with 2x, 0.5x, and 0.2x sodium citrate powder, the cells were incubated with the blocking solution at 37° for 30 minutes. Then, the cells were incubated with biotinylated murine anti-digoxin antibodies for 2 hours at room temperature. The cells were washed three times with PBS and subsequently incubated with secondary antibody (H + L) DyLight594-conjugated AffiniPure goat anti-mouse IgG (Boster, 1:200). After washing three times, anti-fluorescence quenching mounting medium (including DAPI) was added to each well and the cells were mounted with cover glass. Finally, the cells were viewed and images were captured under a ZEISS LSM800 confocal microscope (Carl Zeiss AG, Germany).

### CpG/IL-10 antibody-induced HLH model

Eight-week-old C57BL/6 J mice received an intraperitoneal (i.p.) injection of CpG at a dose of 50 μg per mouse and 0.2 mg anti-mouse IL-10R antibody per mouse on Day 0, Day 2, Day 4, and Day 7 for a total of four times. Then, circMETTL3-NC and circMETTL3-ASO were administered via the tail vein at a dose of 10 mg per mouse on Day 4 and Day 7. The negative control mice received 200 μL of PBS. On Day 9, the mice were sacrificed, and blood was collected for inflammatory cytokine analysis and routine blood tests with parameters including red blood cell (RBC), white blood cell (WBC), platelet (Plt), and hemoglobin (Hb) counts. Important organs, including livers and spleens, were obtained and weighed. Blood was collected and centrifuged, and RBCs in the precipitate were lysed with RBC lysis buffer. Then, the cells were resuspended in FACS buffer (PBS containing 2% FBS) and stained for CD11b, CD206 and CD86 to analyze the proportion of M1 macrophages. Finally, spleens and livers were fixed in 4% paraformaldehyde overnight, rinsed in PBS, dehydrated, embedded in paraffin, and processed into sections of 4-μm thickness. The sections were used for immunohistochemistry (IHC) staining of HE and immunofluorescence (IF) staining of F4/80 and iNOS, according to IHC and IF protocols.

### Poly I:C/LPS-induced lethal sHLH model

#### CircMETTL3-ASO for the treatment of sHLH

Eight-week-old C57BL/6 J mice received an intravenous (i.v.) injection of circMETTL3-ASO or control 10 nm for the first time after they were intraperitoneally (i.p.) injected with 10 mg/kg poly I:C. Then, 12 hours later, the mice were challenged with circMETTL3-ASO or control 10 nm for the second time. After the abovementioned treatments 12 hours later, i.p. administration of 4 mg/kg lipopolysaccharide (LPS) was performed.

### Inflammatory model of sHLH exacerbated by peptides

Equal volumes of 8-week-old C57BL/6 J mice received an intraperitoneal (i.p.) injection of 10 mg/kg poly I:C on Day 0. Then, i.p. administration of 4 mg/kg LPS was performed 24 h later. Mice were given METTL3-156aa for the first time 12 hours after poly i: C and (i.v.) and for the second time during LPS administration, with a dose of 200 μg/time.

The above sHLH models were established for 2 independent experiments. One model was monitored over a period of up to 3 days for survival. In another model, the mice were sacrificed 4 hours after all treatments, and blood samples were collected for inflammatory cytokine analysis and routine blood tests with parameters including red blood cell (RBC), white blood cell (WBC), platelet (Plt), and hemoglobin (HgB) counts.

### Statistical analysis

We statistically analyzed the data using GraphPad 8 Prism statistical software. For comparison between two groups, use t-test for data that follows a normal distribution, and use Mann–Whitney *U* test for data that does not follow a normal distribution. Oneway ANOVA followed by post hoc Bonferroni test were used for multiple comparisons.The log-rank Mantel–Cox test was used to compare Kaplan–Meier curves. *p* < 0.05 was considered statistically significant. *p < 0.05, **p < 0.01, ***p < 0.001, ****p < 0.0001.

## Supplementary information

Supplementary material

uncroped western blots

Table S1

Table S2

## Data Availability

The datasets used and/or analyzed during the current study are available from the corresponding author on reasonable request.
