# Supplementary material for: CircMETTL3-156aa reshapes the glycolytic metabolism of macrophages to promote M1 polarization and induce cytokine storms in sHLH
Source: Cell Death Discov. 2024 Oct 9;10:431. doi: 10.1038/s41420-024-02202-0 (PMC11464708; doi:10.1038/s41420-024-02202-0)
Supplement: Supplementary file 1 — Supplementary material [file 41420_2024_2202_MOESM1_ESM.pdf]

Supplementary Materials for

CircMETTL3-156aa reshapes the glycolytic metabolism of macrophages to promote M1 polarization and induce cytokine storms in sHLH

Authors:

Longlong Xie[1,2]; Xiangying Deng[3]; Xiao Li[2,4]; Xun Li[2,5]; Xiangyu Wang[2,5]; Haipeng Yan[2]; Lin Zhao[6]; Dan Yang[5]; Ting Luo[2,5]; Yufan Yang[2]; Zhenghui Xiao[2]*; Xiulan Lu[2]*

Correspondence to: xiaozh888@126.com (Z. Xiao); 13787252674@163.com (X. Lu)

This PDF file includes:

Supplementary Materials and Methods

Supplementary figures S1-S12

Supplementary Materials and Methods

Mass spectrometry analysis

The proteins were separated by SDS-PAGE and visualized by silver stain development. Gel bands were manually cut and digested with sequencing grade trypsin (Promega, Madison, Wisconsin, USA). Digested peptides were separated by chromatography using a nanoliter flow rate Easy nLC 1200 Chromatography System (Thermo Scientific, USA). Peptides were isolated and subjected to analysis on a Q-Exactive mass spectrometer (Thermo Fisher Scientific, Waltham, Massachusetts, USA). The mass spectrometry database search software is MaxQuant 2.0.1.0.The following protein databases were used: uniprot-Reference proteome-Homo sapiens (Human) [9606]-20607-20221014.fasta.

Droplet-based single-cell RNA sequencing (scRNA-seq)

Fresh peripheral blood mononuclear cells (PBMCs) from 4 pediatric patients with HLH were isolated by ficoll gradient separation. Then, the cells were suspended at a density of 1,000 cells/mL in PBS, and about 10,000 cells were loaded onto a Chromium Controller instrument after completion of a cell suspension preparation procedure using GemCode Gel Bead and Chip (10xGenomics) following the manufacturer's recommendations. Libraries were pre pared using 10xGenomics

Library Kits and sequenced on an Illumina NovaSeq 6000 to generate 150-bp paired-end reads according to the manufacturer's instructions (Berry Genomics).

Preprocessing of scRNA-seq data

The scRNA-seq datas of health controls come from https://doi.org/10.1038/s41467-021-25771-5. The data processing steps you described were carried out using CellRanger (version 6.0.1, 10x Genomics) for alignment and quantification, and the R package Seurat (version 4.1.0) for quality control filtering, variable gene selection, dimensionality reduction, and cell clustering. For further analysis of cell populations, only cells with less than 7.5% mitochondrial genes were kept.To avoid batch effects, the "FindIntegrationAnchors" function was applied to integrate the samples. Normalization, scaling, and variable gene selection were performed using the "NormalizedData," "FindVariableFeatures," and "ScaleData" functions in Seurat with standard settings. In addition, the R package "scDblFinder" was used to identify doublets.

Single-cell RNA sequencing (scRNA-seq) raw reads from PBMCs of HLH patients were mapped to the human hg38 genome using CellRange 6.1.2 (ref1). As a control, scRNA-seq data from PBMCs of healthy children were obtained from the Gene Expression Omnibus (GEO) database under accession number GSE168732 (ref2). Quality control and data analysis were performed using the Seurat 5.0.1 package in R (ref3). Doublets were filtered out using scDblFinder 3.16 (ref4) with the expected doublet rate of 0.1. Data were normalized and scaled by the NormalizeData and ScaleData functions. The top 2000 variable genes were selected by the FindVariableFeatures function for the dimensionality reduction. The top 30 principle components were computed by the RunPCA function. Batch effects among samples was removed with the CCAIntegration method. The top 30 cca dimensions were used for the UMAP calculation using RunUMAP. Cells were unsupervised clustering into cell populations using FindClusters at a resolution of 0.4. The celltype of each cluster were determined by its marker genes. Propotion of each celltype were quantified and compared statistically using t-test.

RNA immunoprecipitation (RIP)

The RIP assay was conducted using the Magna RIP RNA-bing Protein Immunoprecipitation kit (BersinBio TM, China) according to the provider's protocol. In brief, cell lysates were cultured with Dynabeads-coated IgG antibody (Cell signaling technology, USA) or SRSF10 antibody (proteintech, China) for 16h at 4°C.

After washing and elution, RNA was extracted, then purified and subjected to qRT-PCR to detect the enriched pre-METTL3 mRNA.

Reagents and antibodies

BE0050 InVivoMAb anti-mouse IL-10R(CD210); CpG ODN 1826 (Integrated DNA Technologies) (The sequence is as follows ： T*C*C*A*T*G*A*C*G*T*T*C*C*T*G*A*C*G*T*T); Poly (I:C) (HMW) (InvivoGen, tlrl-pic-5); Regarding the primary antibodies used in the study, anti-GAPDH; anti-METLL3-156aa was purchased from GL.Biochem (Shanghai, China); anti-LDHA, anti-SRSF10, anti-F4/80, anti-CD206, anti-CD86, anti-CD11b, anti-CD56 were purchased from Cell Signaling Technology (Danvers, PA USA). All details are available from Table S2.

Extracellular acidification rate (ECAR)

ECAR was measured using the glycolysis stress kit (Luxcel) and analyzed by the XF96 Extracellular Flux analyzer (Seahorse Bioscience). Glycolysis, glycolytic capacity and glycolytic reserve were determined by the sequential addition of 10 mM glucose, 1 μM oligomycin and 50 mM 2-D-glucose. ECAR was normalized to cell protein concentration in each experiment.

LDHA enzyme activity

LDHA enzyme activity assay was obtained according to the Lactate dehydrogenase (LDH) activity assay kit instructions (solarbio life science). Briefly, 20 μl of extract was added to $1.0x10^5$ cells or cells were crushed by ultrasound (ice bath, power 20% or 200W, sonication 3s, interval 10s, repeated 30 times). Subsequently, the supernatant was centrifuged at 8000g for 10min at 4℃, and the LDHA enzyme activity detection reagent was added and incubated at room temperature, and the absorbance value was read at 450nm. Definition of unit: 1 nmol of pyruvate per 10,000 cells per minute of catalytic production is defined as one unit of enzyme activity.

Immunohistochemistry

The tissue sections were baked in a 60°C thermostat for 60 minutes, then immediately placed in environmentally friendly wax dewaxing, sequentially soaked in anhydrous ethanol, 85% ethanol, and 75% ethanol for 5 minutes each, and double-distilled water rinsed for 1 minute for permeabilization. Subsequently, the sections were placed in a sodium citrate buffer solution for repair. Anti-inflammatory and fixed tissues were protected by a 3% H_2O_2 solution configured with methanol.

Closure of the remaining sites on the tissue sections that bind non-specifically to the primary antibody was performed with a serum homologous to the secondary antibody. Next, primary antibody 1:100 was left overnight. After binding the secondary antibody, Diaminobenzidine (DAB) color development and hematoxylin stained nuclei can be immersed in water to return to blue. The sections were dried and sealed with neutral resin. Immunohistochemical scoring criteria were: (1) Positive signal intensity: 0 points for colorless, 1 point for light yellow, 2 points for brown, and 3 points for tan or brown. (2) Percentage of positive cells: 1 point for positive cell rate less than or equal to 10%, 2 points for positive cell rate greater than or equal to 50%, 3 points for positive cell rate greater than or equal to 50% and less than or equal to 75%, and 4 points for positive cell rate greater than or equal to 75%. (3) Then the positive signal intensity and the score of positive rate were multiplied to get the final score. The results were analyzed using GraphPad 8 software.

Immunoprecipitation (Co-IP)

After overexpression of circMETTL3 for 48 hours, 293T or THP-1 cells were collected and lysed to obtain the protein solution, and two copies of 1 mg protein were taken, i.e., experimental group and IgG negative control group. Then, 15 μl protein A/G was added separately to preclear the non-specific binding molecules from the protein for 2h. Discard the magnetic beads and add flag or IgG (negative control) antibodies to the above proteins, respectively, and bind overnight at 4°C. Add 20 μl protein A/G to the protein-antibody mixture and combine at 4°C for 2 h. Discard the supernatant and wash protein A/G three times with ice-cold PBS for 10 min each time. Resuspend the magnetic beads with 20 μL of 1×Loading Buffer, mix well, 100°C, 5 min to dissociate the protein complex on the beads. Centrifuge at 13000 rpm for 5 min and transfer the supernatant to a new EP tube. The samples were subjected to SDS-PAGE gel electrophoresis with input as the positive control and IgG group as the negative control.

Polysome Fractionation by Sucrose Gradients

Separated spermatocytes were homogenized in lysis buffer (100 mM NaCl, 10 mM MgCl2, 30 mM Tris-HCl [pH 7.5], 1 mM DTT, 30 U/ml RNasin) supplemented with 1% Triton X-100. The lysate was incubated on ice for 5 min and then centrifuged at 12,000 g for 10 min at 4° C. The supernatant was obtained and slowly added on a 15-50% (wt/vol) sucrose gradient and centrifuged for 110 min at 37,000 rpm/min in an ultraspeed centrifuge. Eleven fractions were collected from top to bottom, and

RNA was extracted into qPCR to analyze the expression of circMETTL3.

Supplementary Figures

Figure S1

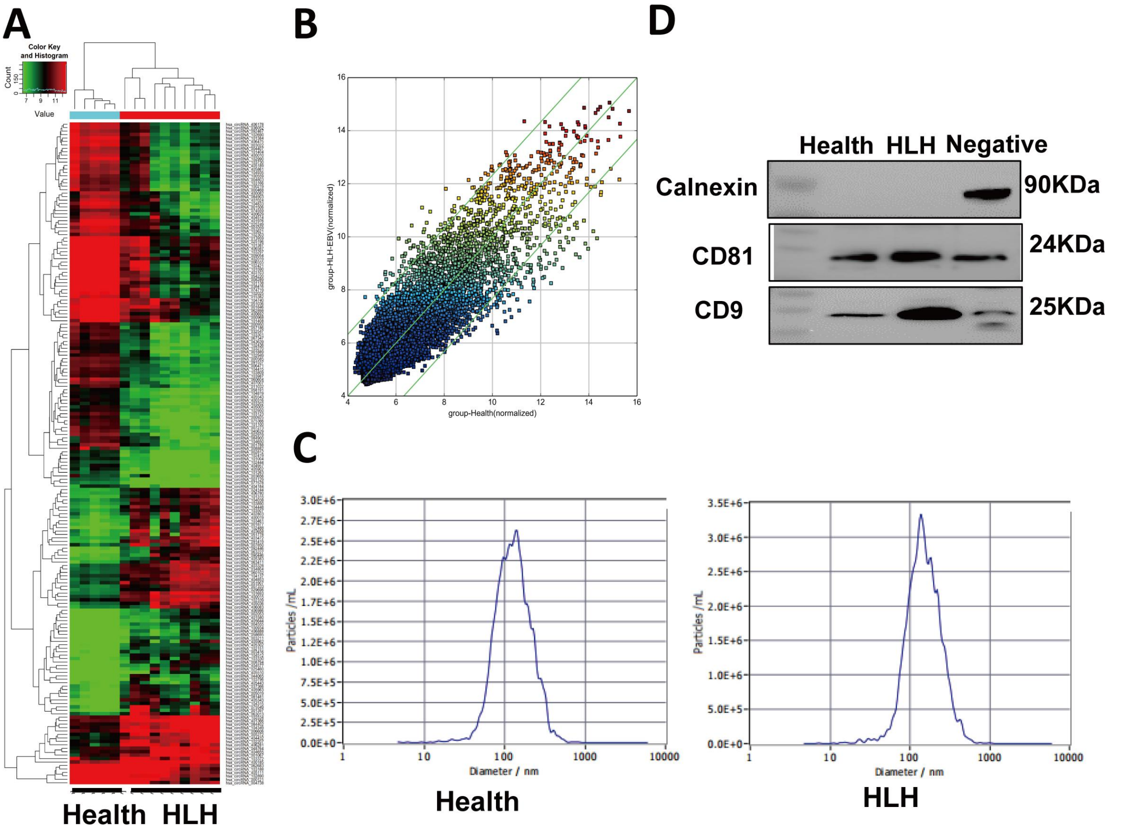

SFigure.1 Supplementary for figure 1

A. Hot map exhibit the differential expression circular RNAs by Circular RNA chip analysis. ($p < 0.01$ and fold change > 5)

B．Scatter plot react the distribution of circRNAs.

C. Identify the concentration of exosomes by NTA.

D．The expression of positive protein CD81, CD9 and negative protein calnexin in health and HLH plasma exosome by western blot.

Figure S2

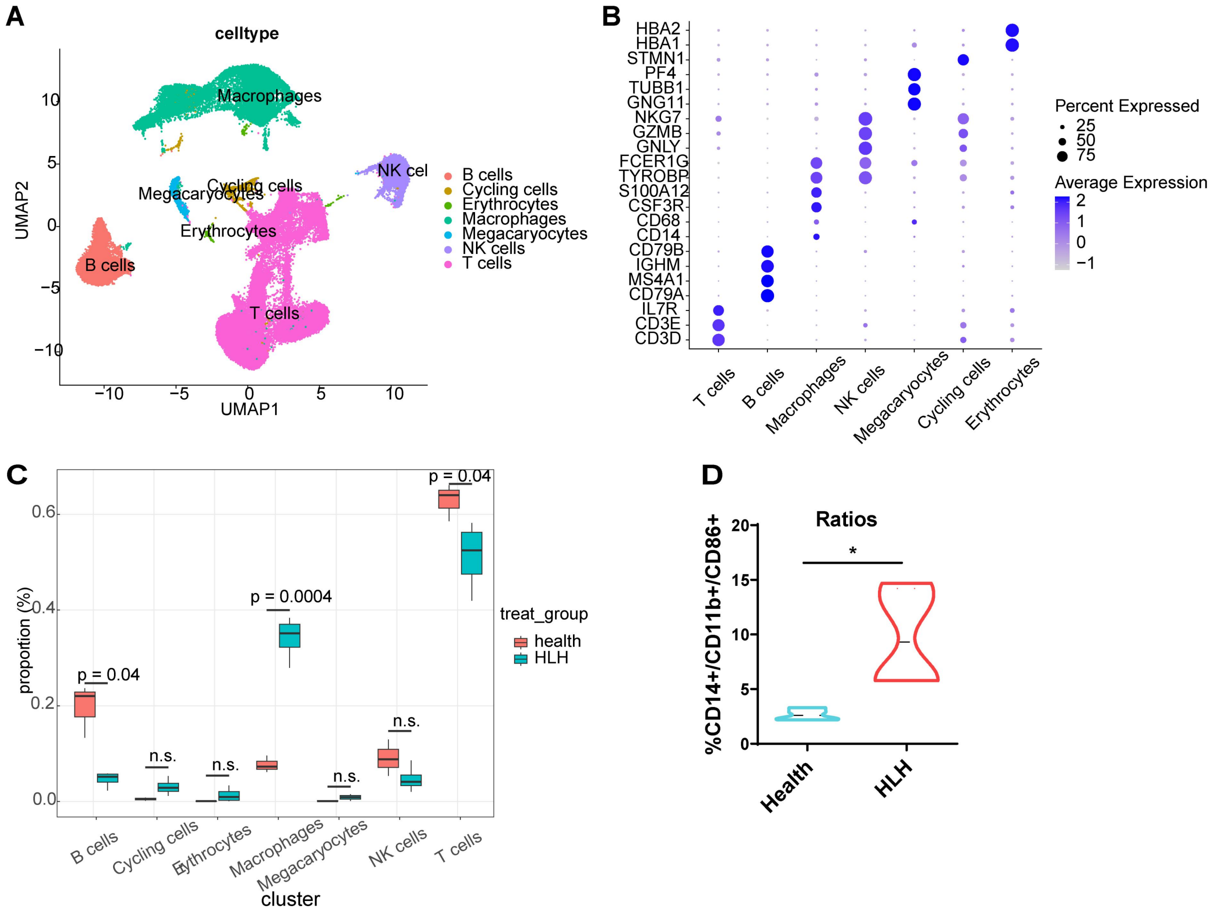

SFigure.2 Activated M1 macrophages in peripheral blood of patients with sHLH

A. UMAP plot of 176,447 single cells grouped into seven major cell types (n = 7).

B. The normalised expression of marker genes for each cell type.

C. Box plots showed the proportion of all cell types (Health, n=3 independent samples, HLH, n=4 independent samples). Data have been represented as Median, n.s., not significant.

D. FACS analysis of CD86+ macrophages ratios in blood from sHLH pediatric patients. Data have been represented as Median (Health, n=4 independent samples, sHLH, n=4 independent samples).

Figure S3

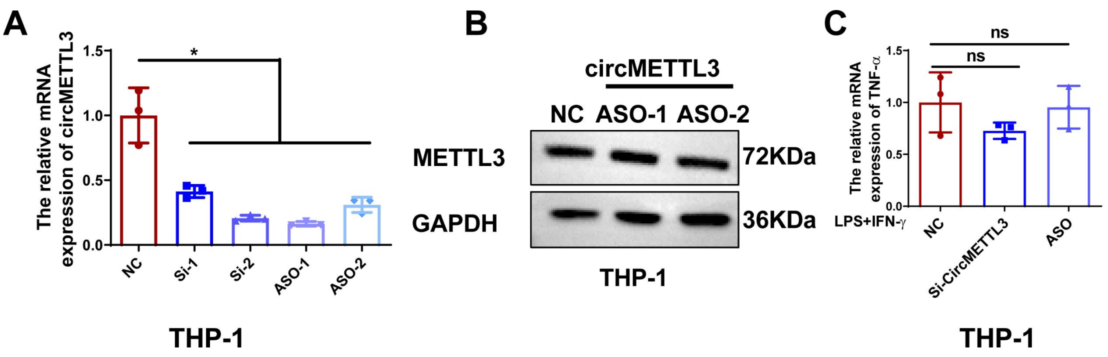

SFigure.3 The efficiency of circMETTL3 knockdown

A. SiRNA and ASO specific target to knock down circMETTL3. Data represent the mean ±SD (n =3 independent experiments, *p<0.05).

B. The level of full lenth METTL3 was examined by western blot in THP-1 cells after circMETTL3 konock down.

C. Changes in intracellular TNF-α levels upon knockdown of circMETTL3 were observed in LPS/IFN-γ treatment. Data represent the mean ±SD (n =3 independent experiments, n.s., not significant).

Figure S4

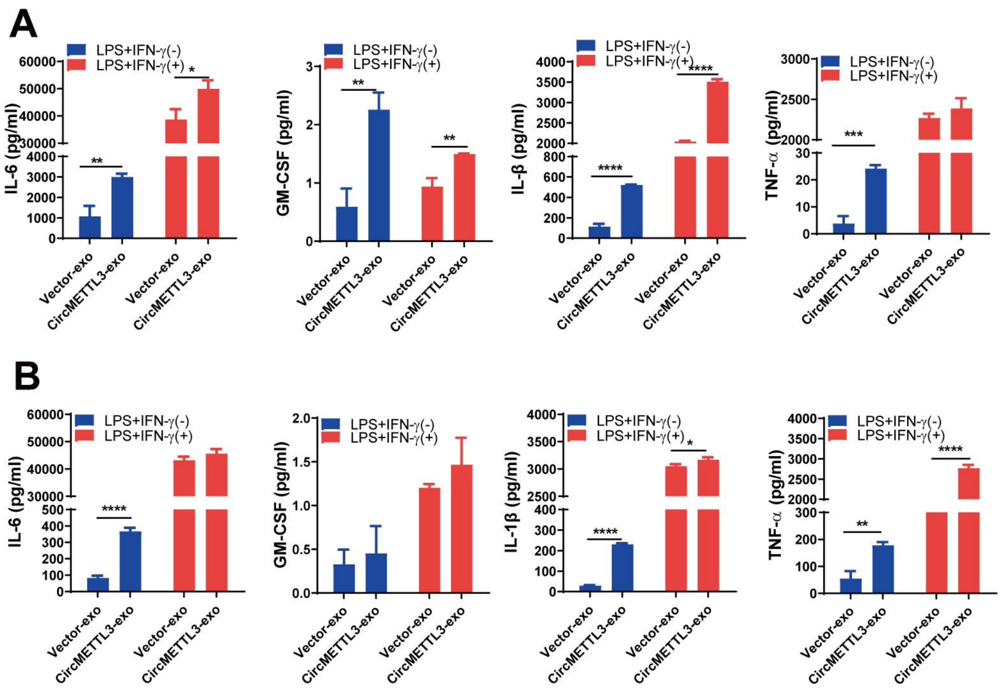

SFigure.4 Supplementary for figure 2

A. Study the various cytokine changes in THP-1 cells stimulated by exosomes in the supernatant of HEK293T cells transfected with circMETTL3 plasmid, with or without treatment of LPS/IFN-γ.

B. Investigation of various cytokines change in THP-1 cells stimulated by THP-1 cell supernatant exosomes with circMETTL3 plasmid transfected, regardless of the presence or absence of LPS/IFN-γ treatment. Error bars represent mean ± SD from n=3 independent experiments, * $p<0.05$, ** $p<0.01$, *** $p<0.001$, **** $p<0.0001$ (**A-B**).

Figure S5

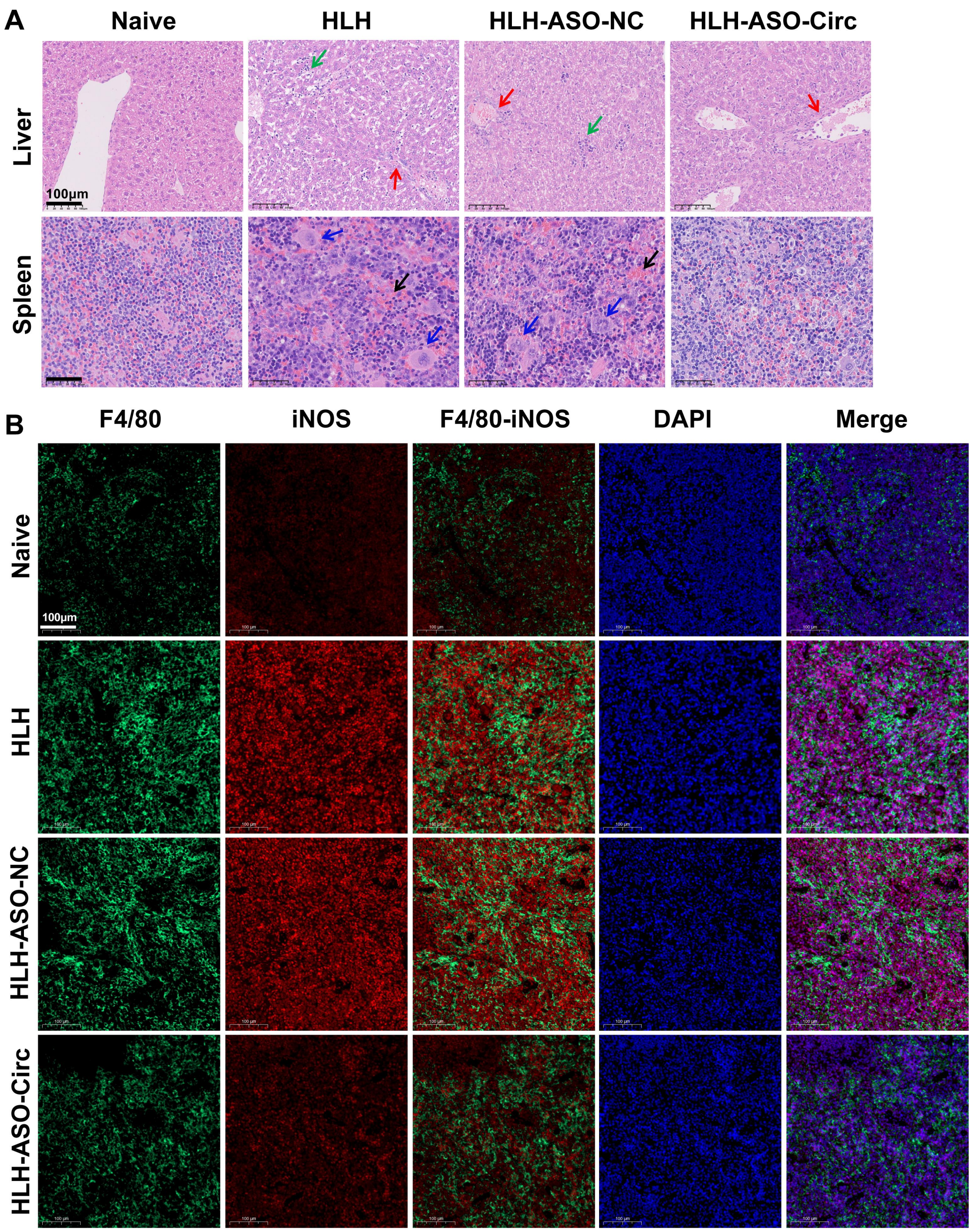

SFigure.5 Supplementary for figure 3

A. Livers and Splenics were stained with H&E in different mouse groups including saline, HLH, HLH-ASO-NC and HLH-ASO-CircMETTL3 (Red arrow, Hepatic portal inflammation; Green arrow, lobular inflammation; Black arrow, hemorrhagic focus; Blue arrow, hemophagocyte; Scale bar, 100μm).

B. Splenic M1 polarization of macrophages were stained by immunofluorescence for macrophage (F4/80) and M1 macrophage (iNOS) markers (Scale bar, 100μm).

Figure S6

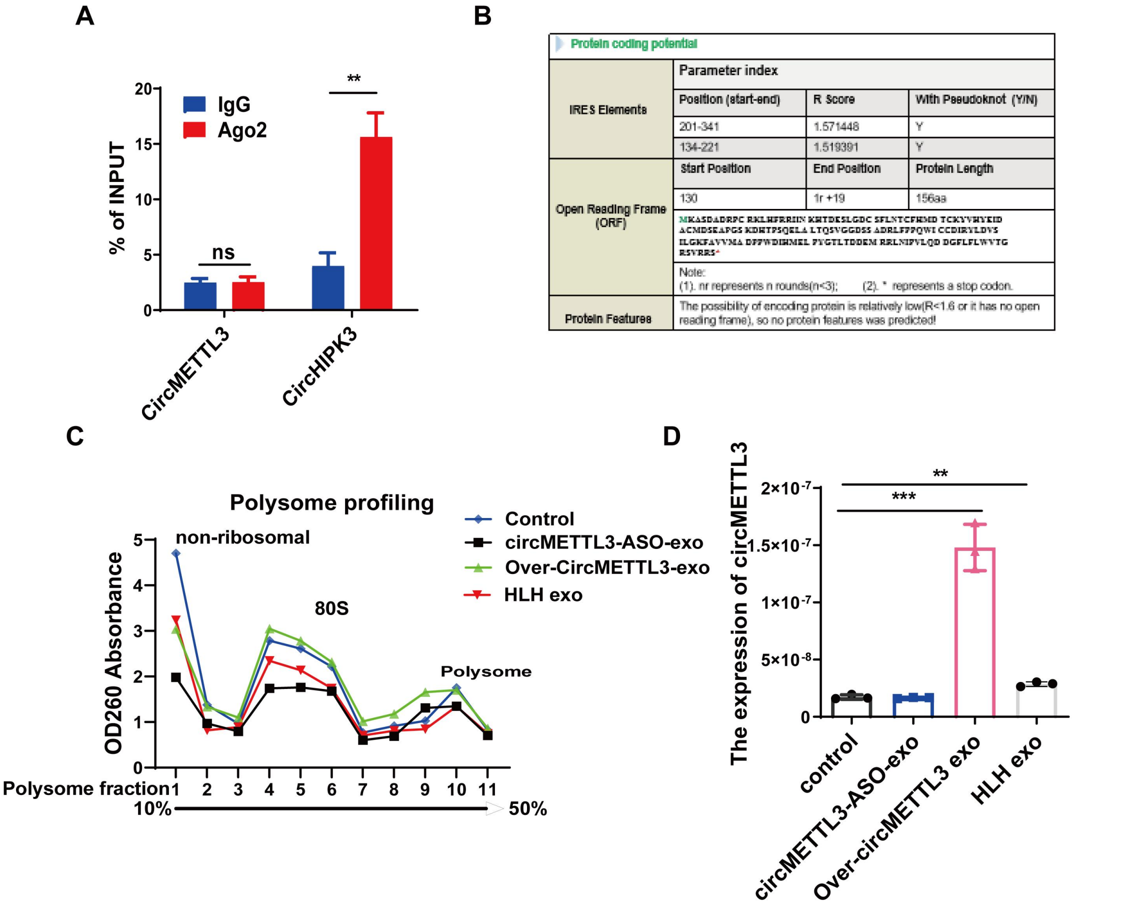

SFigure.6 Analysis the potential of circMETTL3 in encoding peptides

A. Anti-AGO2 antibody was used for RIP analysis of circMETTL3. The qPCR to detect the enrichment of RIP RNA, with each value normalized to the input RNA levels used in the RIP analysis. CircHIPK3 serves as a positive control. Data represent the mean ±SD (n =3 independent experiments, ** $p < 0.01$, n.s., not significant).

B. Predicting the coding potential of circMETTL3 using the circRNADb website.

C. Polysome profiling of THP-1 cells stimulated by HLH-derived exosomes and 293T cell supernatant exosomes with circMETTL3-overexpression or circMETTL3-ASO plasmid transfected.

D. Relative enrichment of circMETTL3 in polysome part (fraction 11) was measured by qRT-PCR. Data represent the mean ±SD (n =3 independent experiments, ** $p < 0.01$, *** $p<0.001$).

Figure S7

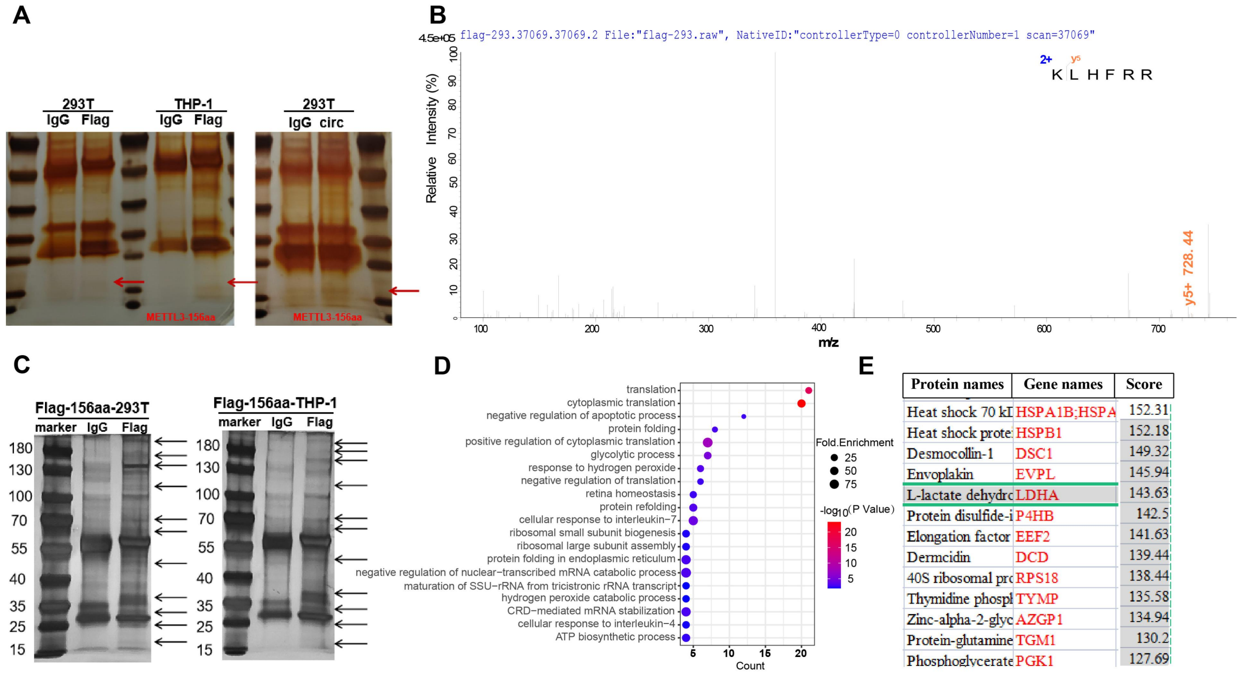

Protein names	Gene names	Score
Heat shock 70 kD	HSPA1B;HSPA	152.31
Heat shock prote	HSPB1	152.18
Desmocollin-1	DSC1	149.32
Envoplakin	EVPL	145.94
L-lactate dehydro	LDHA	143.63
Protein disulfide-i	P4HB	142.5
Elongation factor	EEF2	141.63
Dermcidin	DCD	139.44
40S ribosomal pro	RPS18	138.44
Thymidine phospl	TYMP	135.58
Zinc-alpha-2-glyc	AZGP1	134.94
Protein-glutamine	TGM1	130.2
Phosphoglycerate	PGK1	127.69

SFigure.7 Supplementary for figure 4

A-B. Immunoblot (IB) and mass spectra (MS) identification of METTL3-156a.a. in 293T cells with METTL3-156aa Flag or circMETTL3 overexpression (OV).

C. Immunoblot (IB) presents proteins interacting with METTL3-156aa.

D. Mass spectrometry (MS) screening for major signaling pathways enriched in proteins interacting with METTL3-156 aa.

E. LDHA was screened for the protein interacting with METTL3 in the glycolytic pathway.

Figure S8

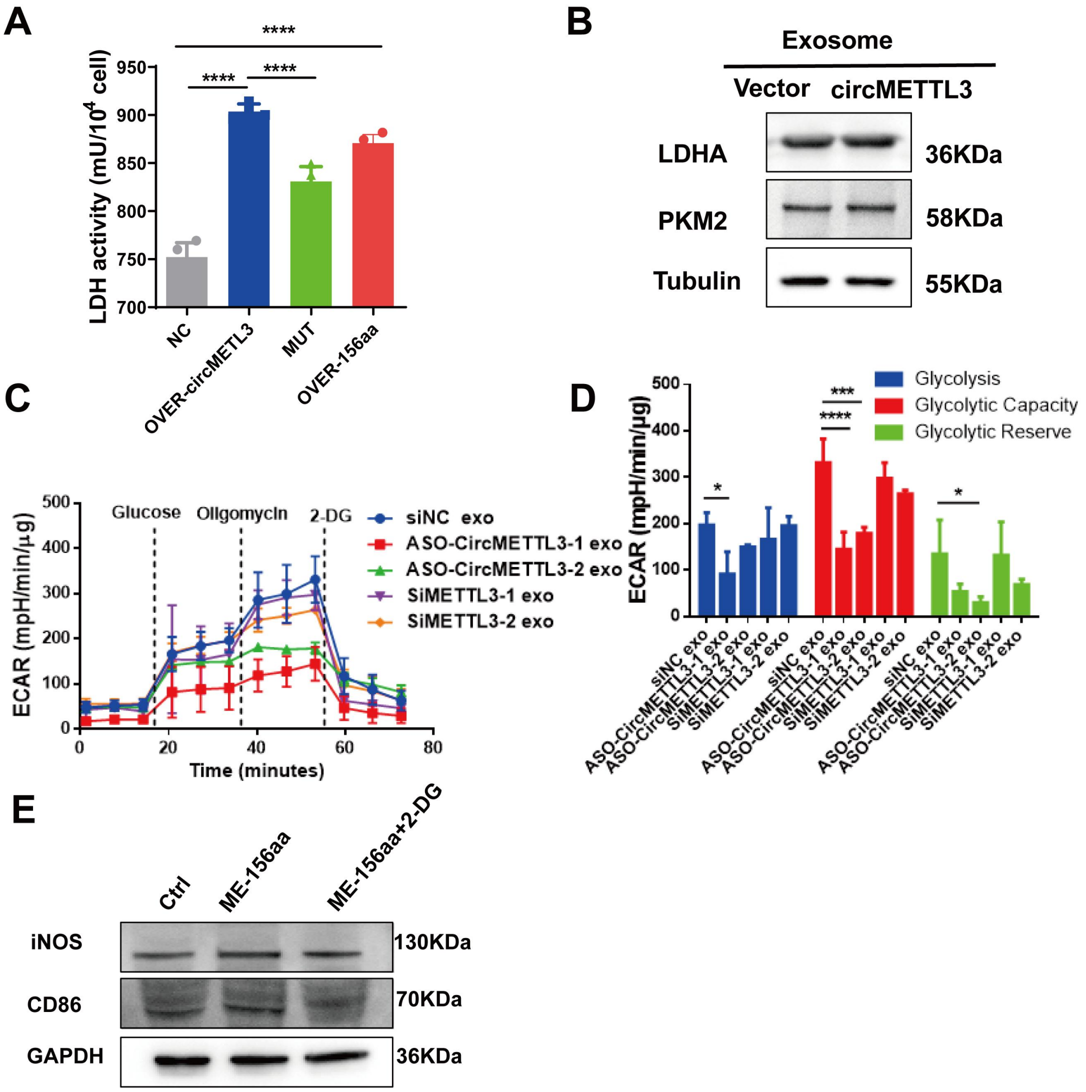

SFigure.8 Exosomal circMETTL3 enhance glycolytic metabolism.

A. LDH actvity was detected in THP-1 cells transfected with Empty Vector, circ-METTL3-3XFlag vector, splicing donor site mutant vector (circMETTL3 mut-3XFlag), and linearized METTL3-156a.a.-3XFlag vector. Data represent the mean ±SD (n =3 independent experiments, **** p<0.0001).

B. The level of LDHA and PKM2 were examined by western blot in THP-1 cells after exosomal circMETTL3 treated.

C-D. ECAR was determined using the Seahorse XF96 analyzer to evaluate the glycolytic flux of THP-1 cells stimulated by extracellular vesicles after circMETTL3 or METTL3 knockout. Glycolysis, glycolytic capacity, and glycolytic reserve were determined by the sequential addition of 10 mM glucose, 1 mM oligomycin, and 50 mM 2-D-glucose. Values represent the mean ± SD of at least three independent experiments (* $p < 0.05$, ** $p < 0.01$, *** $p < 0.001$).

E. Expression of iNOS and CD86 in PMA-pretreated THP-1 cells treated with the peptide METTL3-156aa alone or in combination with 2-DG was examined by Western blotting.

Figure S9

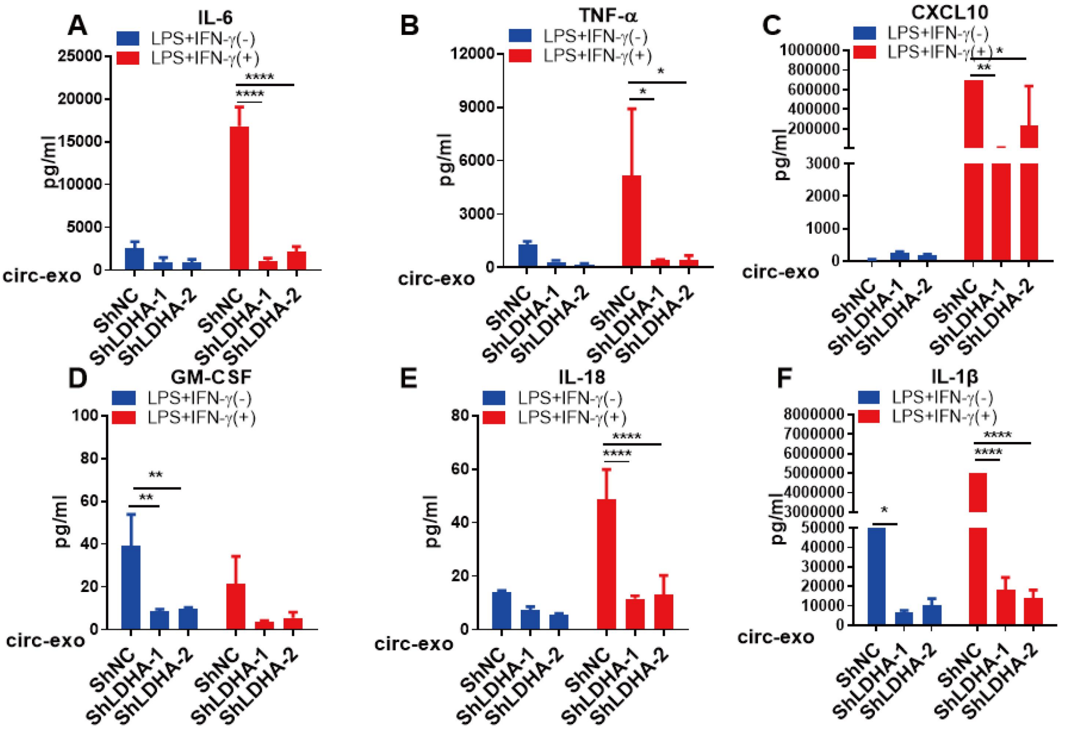

SFigure.9 The role of LDHA in activating M1 macrophages with exosomal circMETTL3. The cytokines level (IL-6, TNF-α, CXCL10, GM-CSF, IL-10, IL-18, IL-1β,) was analyzed in THP-1 cells treated with exosomal circMETTL3 in the presence of shRNA LDHA (A-F). Data represent the mean ±SD (n =3 independent experiments, * $p < 0.05$, ** $p < 0.01$, *** $p < 0.001$, **** $p<0.0001$).

Figure S10

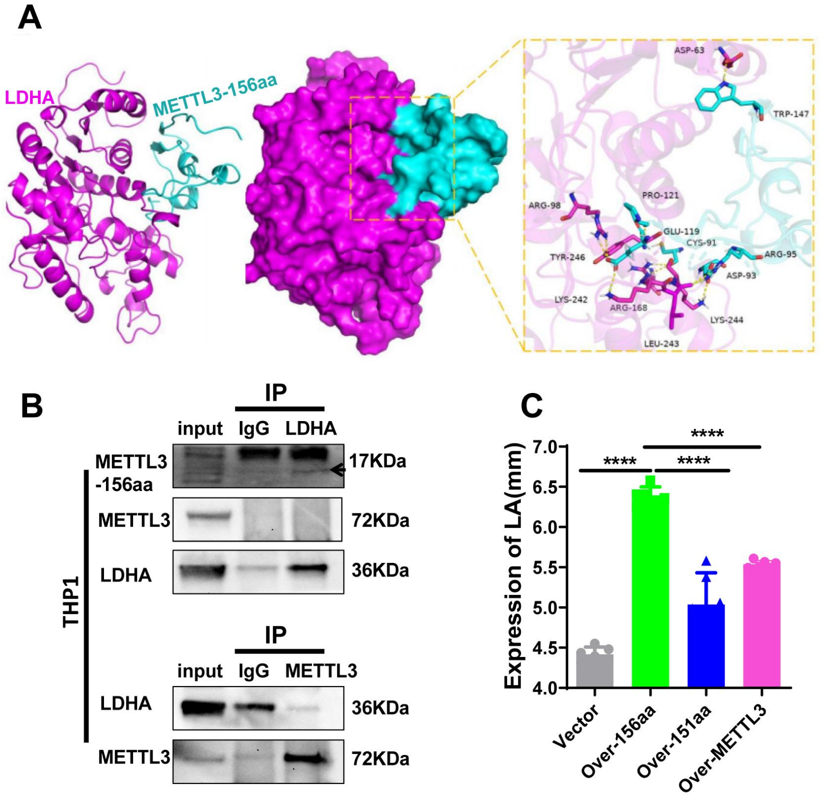

SFigure.10 Supplementary for figure 6

A. Use HDOCK software for the docking study of METTL3-156aa and LDHA.

B. Mutual interaction of LDHA and METTL3 in HEK293T and THP-1 cells were determined by IP.

C. Lactate levels of THP-1 cell supernatants after overexpression with Empty Vector, METTL3-156aa vector, SVRRS site-mutated METTL3-156aa vector and METTL3 vector. Data are shown as mean ±SD (n =3 independent experiments, **** *p*<0.0001).

Figure S11

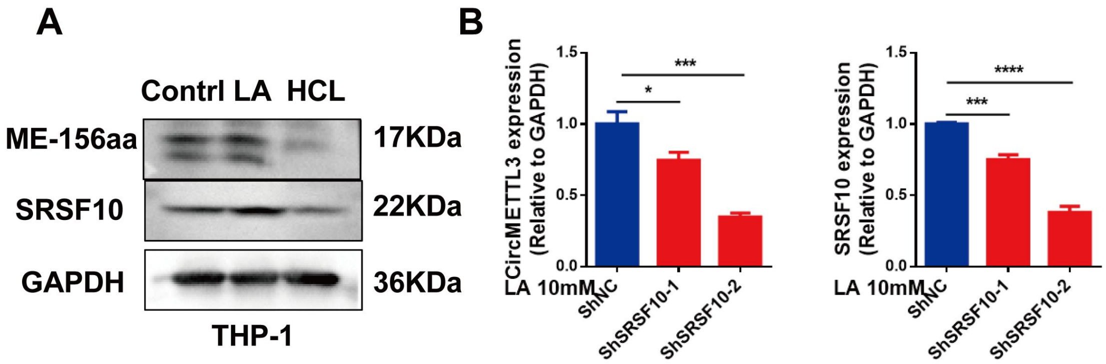

SFigure. 11 Lactic acid regulates the generation of circMETTL3 through SRF10

A. Western blot show the protein level of METTL3-156aa and SRSF10 after treat with lactate or hydrochloric acid.

B. The expression of circMETTL3 of SRSF10 knockout THP-1 cells with the lactic acid treated. Data are shown as mean ±SD (n =3 independent experiments, * $p < 0.05$, *** $p < 0.001$, **** $p<0.0001$).

Figure S12

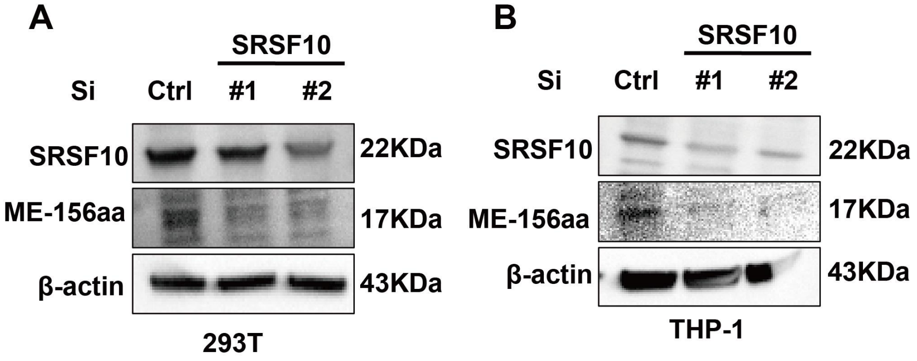

SFigure. 12 The efficiency of SRSF10 knock down

A-B. Western blot show the level of METTL3-156aa by Knock down SRSF10.
