# Supplementary material for: CircMETTL3-156aa reshapes the glycolytic metabolism of macrophages to promote M1 polarization and induce cytokine storms in sHLH
Source: Cell Death Discov. 2024 Oct 9;10:431. doi: 10.1038/s41420-024-02202-0 (PMC11464708; doi:10.1038/s41420-024-02202-0)
Supplement: Supplementary file 2 — uncroped western blots [file 41420_2024_2202_MOESM2_ESM.pptx]

## Slide 1
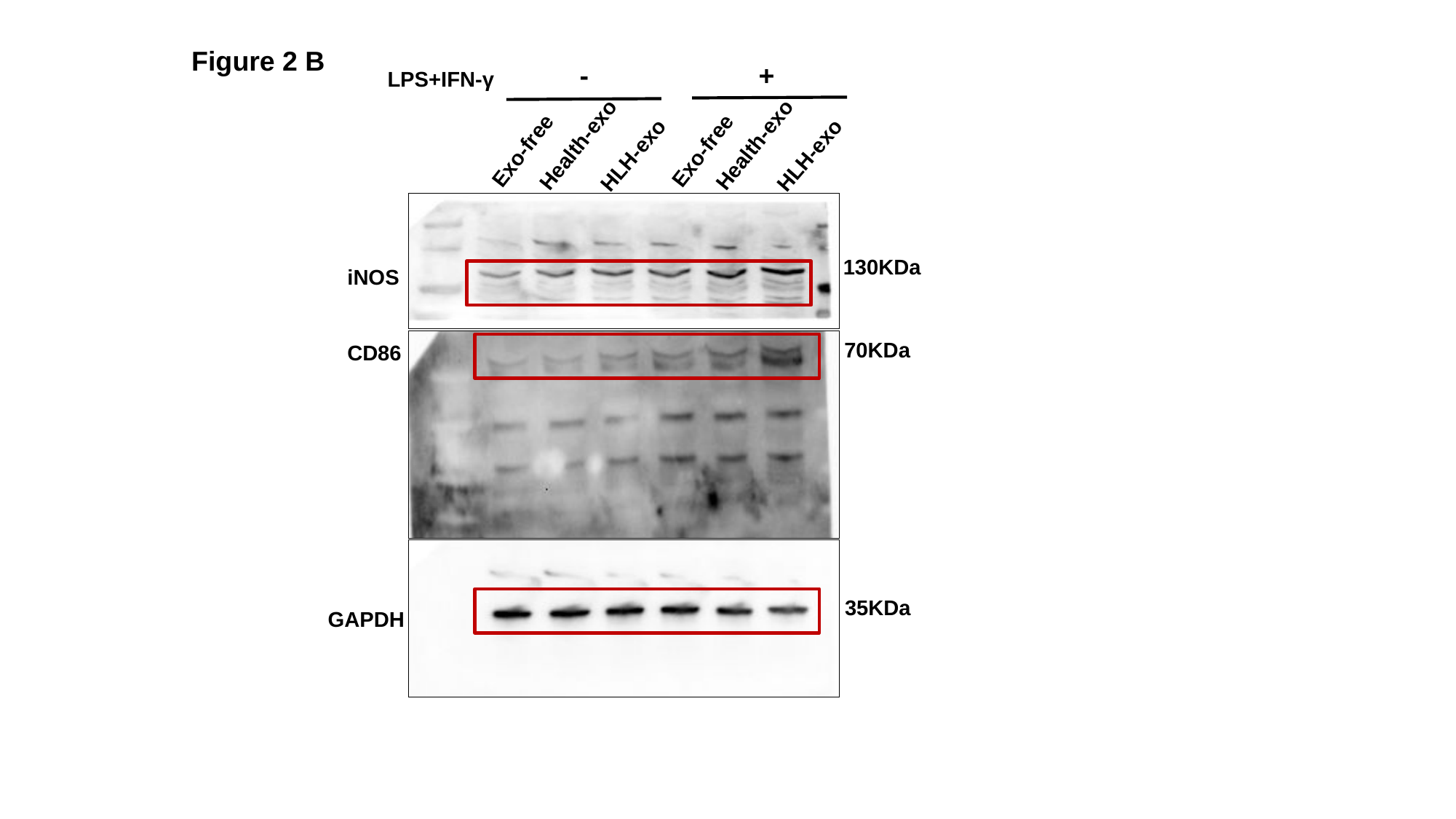

Figure 2 B
-
+
LPS+IFN-γ
 Health-exo
 Health-exo
Exo-free
Exo-free
HLH-exo
HLH-exo
130KDa
iNOS
70KDa
CD86
GAPDH
35KDa
THP-1

## Slide 2
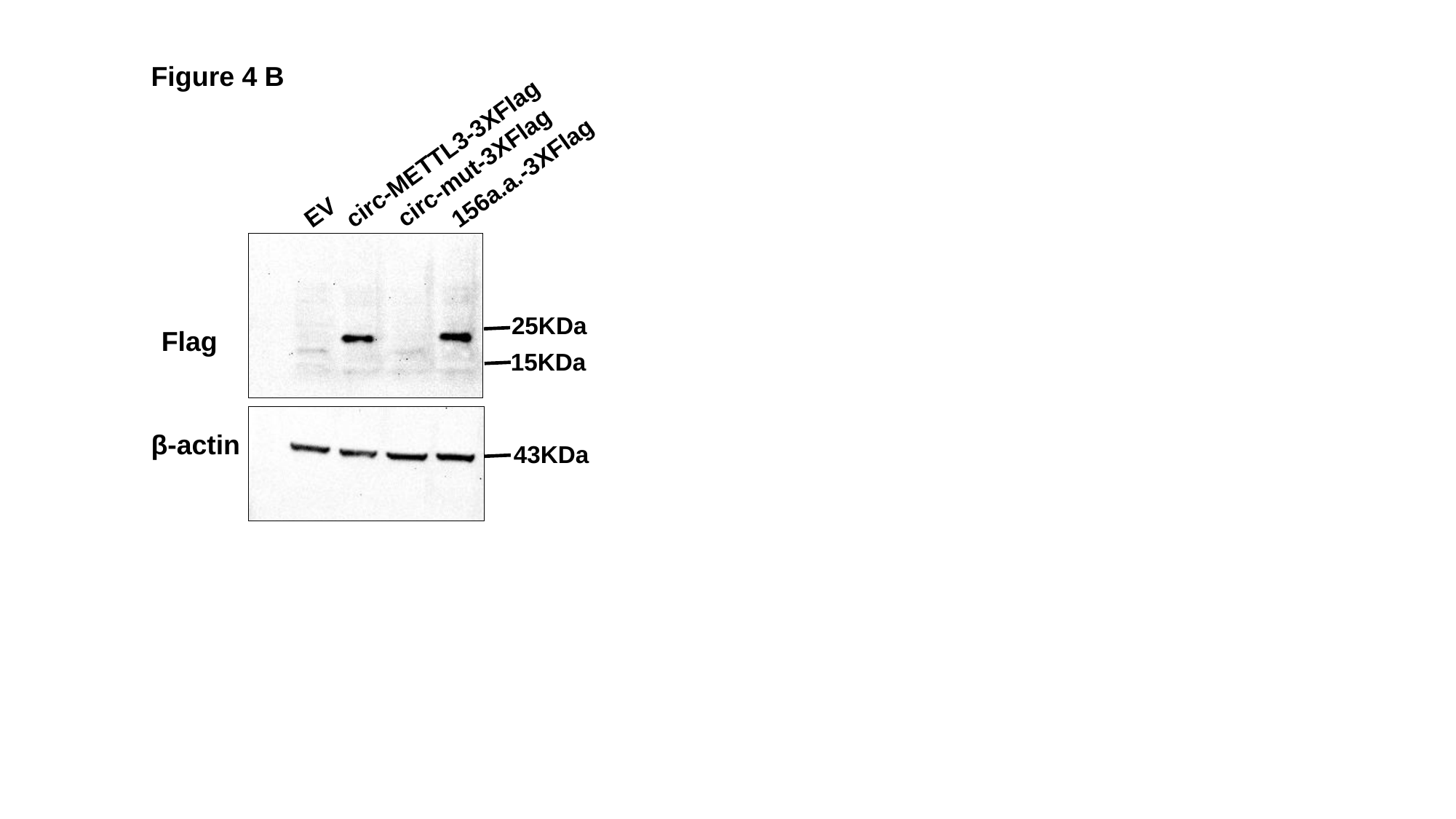

Figure 4 B
circ-mut-3XFlag
circ-METTL3-3XFlag
156a.a.-3XFlag
EV
25KDa
Flag
15KDa
β-actin
43KDa

## Slide 3
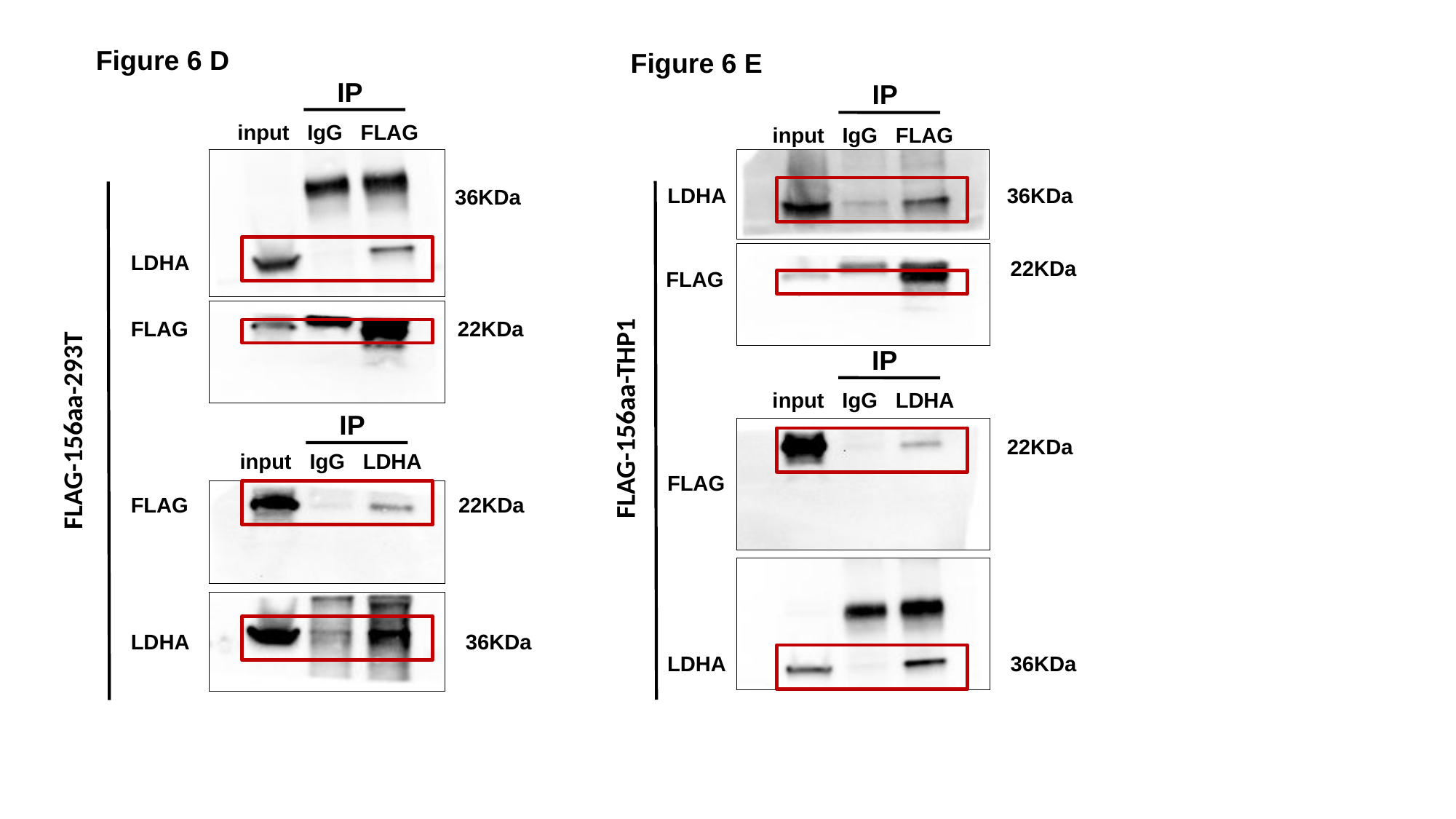

Figure 6 D
IP
input IgG FLAG
36KDa
LDHA
FLAG
22KDa
FLAG-156aa-293T
IP
input IgG LDHA
FLAG
22KDa
LDHA
36KDa
Figure 6 E
IP
input IgG FLAG
LDHA
36KDa
22KDa
FLAG
IP
input IgG LDHA
FLAG-156aa-THP1
22KDa
FLAG
LDHA
36KDa

## Slide 4
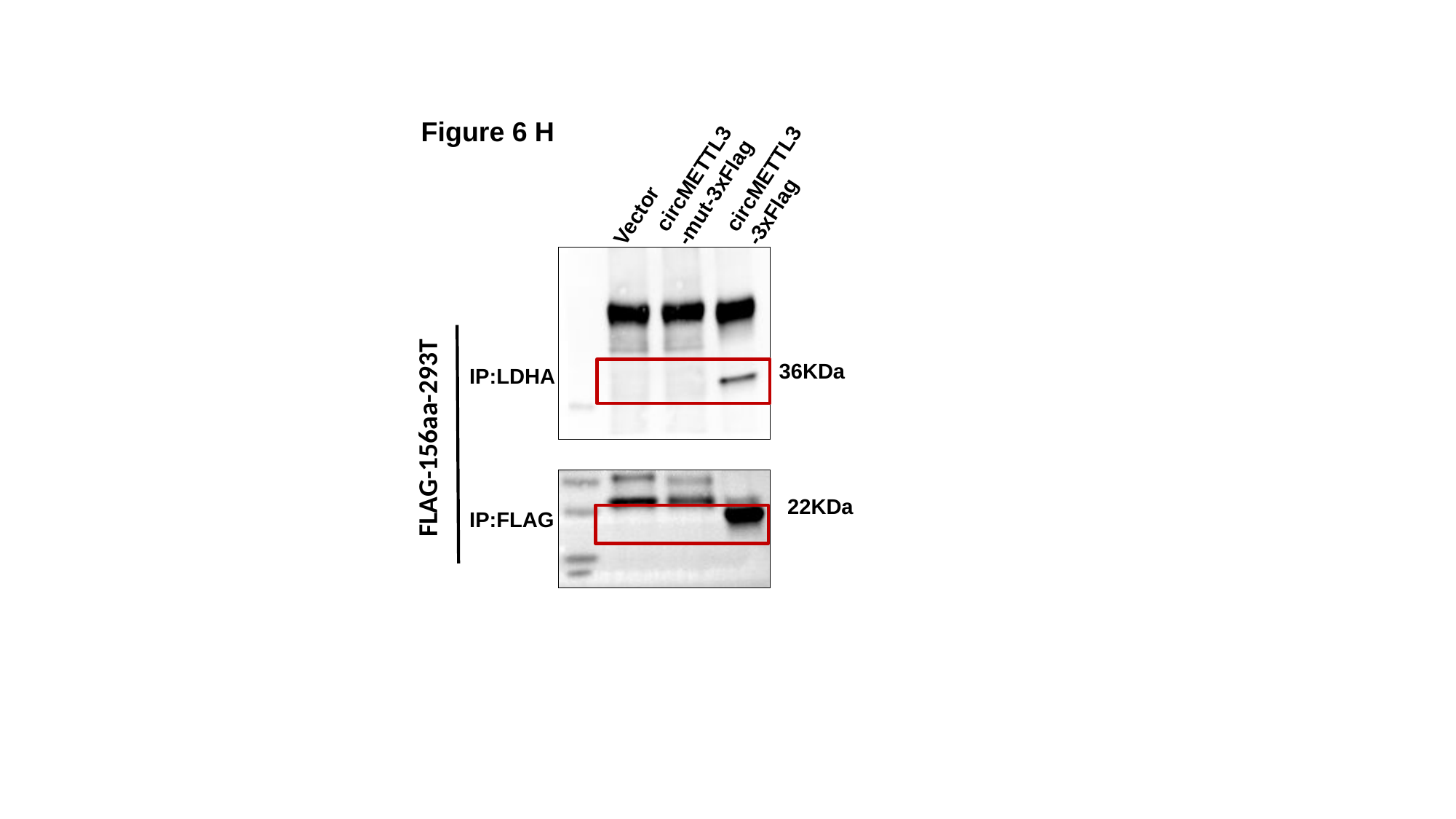

Figure 6 H
circMETTL3
-mut-3xFlag
circMETTL3 -3xFlag
Vector
36KDa
IP:LDHA
FLAG-156aa-293T
22KDa
IP:FLAG

## Slide 5
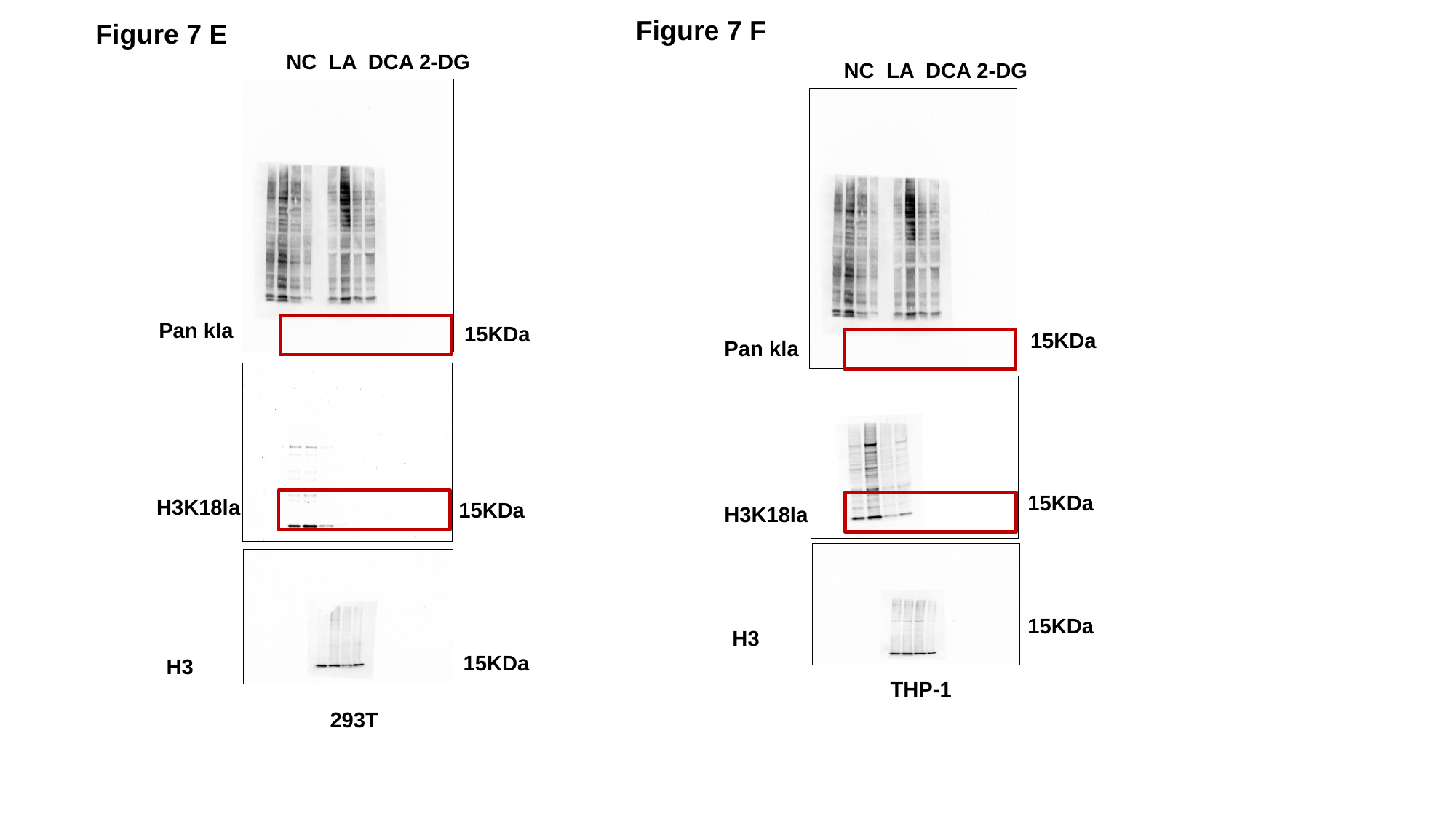

Figure 7 F
 NC LA DCA 2-DG
15KDa
Pan kla
15KDa
H3K18la
15KDa
H3
THP-1
Figure 7 E
 NC LA DCA 2-DG
Pan kla
15KDa
H3K18la
15KDa
15KDa
H3
293T

## Slide 6
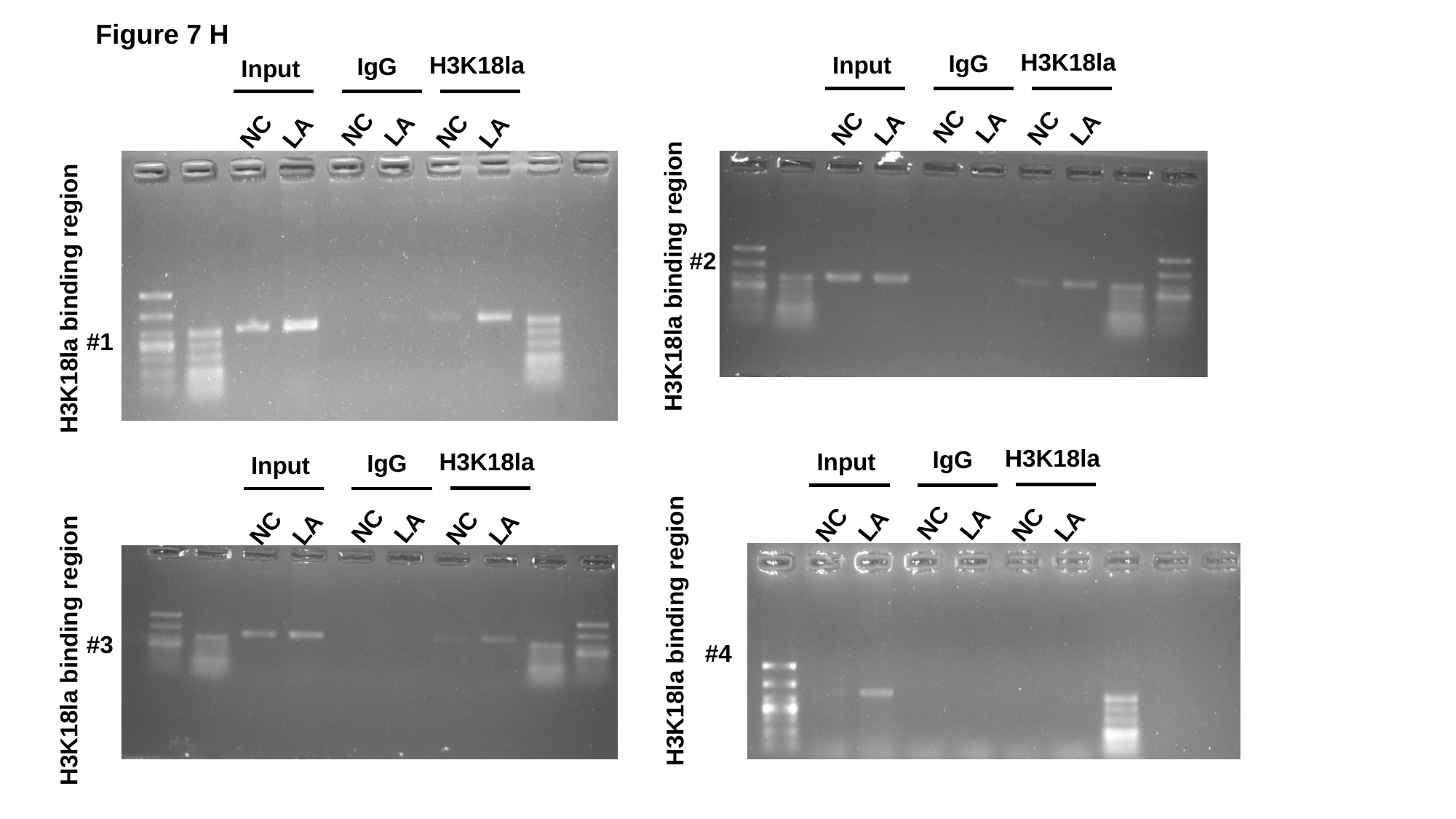

Figure 7 H
H3K18la
IgG
Input
NC
NC
NC
LA
LA
LA
H3K18la
IgG
Input
NC
NC
NC
LA
LA
LA
#2
H3K18la binding region
H3K18la binding region
#1
H3K18la
IgG
Input
NC
NC
NC
LA
LA
LA
H3K18la
IgG
Input
NC
NC
NC
LA
LA
LA
H3K18la binding region
H3K18la binding region
#3
#4

## Slide 7
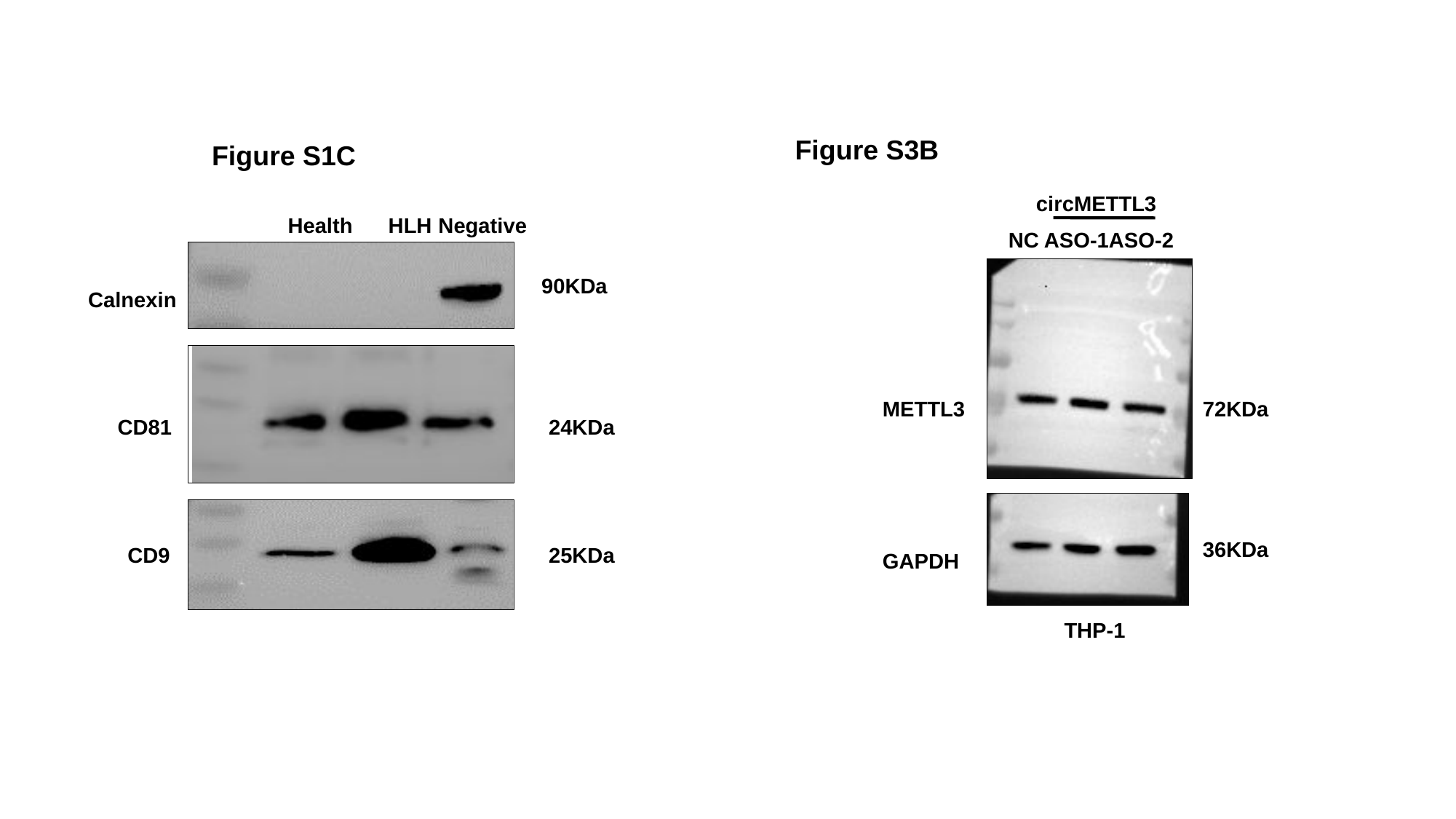

Figure S3B
Figure S1C
 Health
 HLH
Negative
Calnexin
CD81
CD9
90KDa
24KDa
25KDa
circMETTL3
NC ASO-1ASO-2
METTL3
72KDa
36KDa
GAPDH
THP-1

## Slide 8
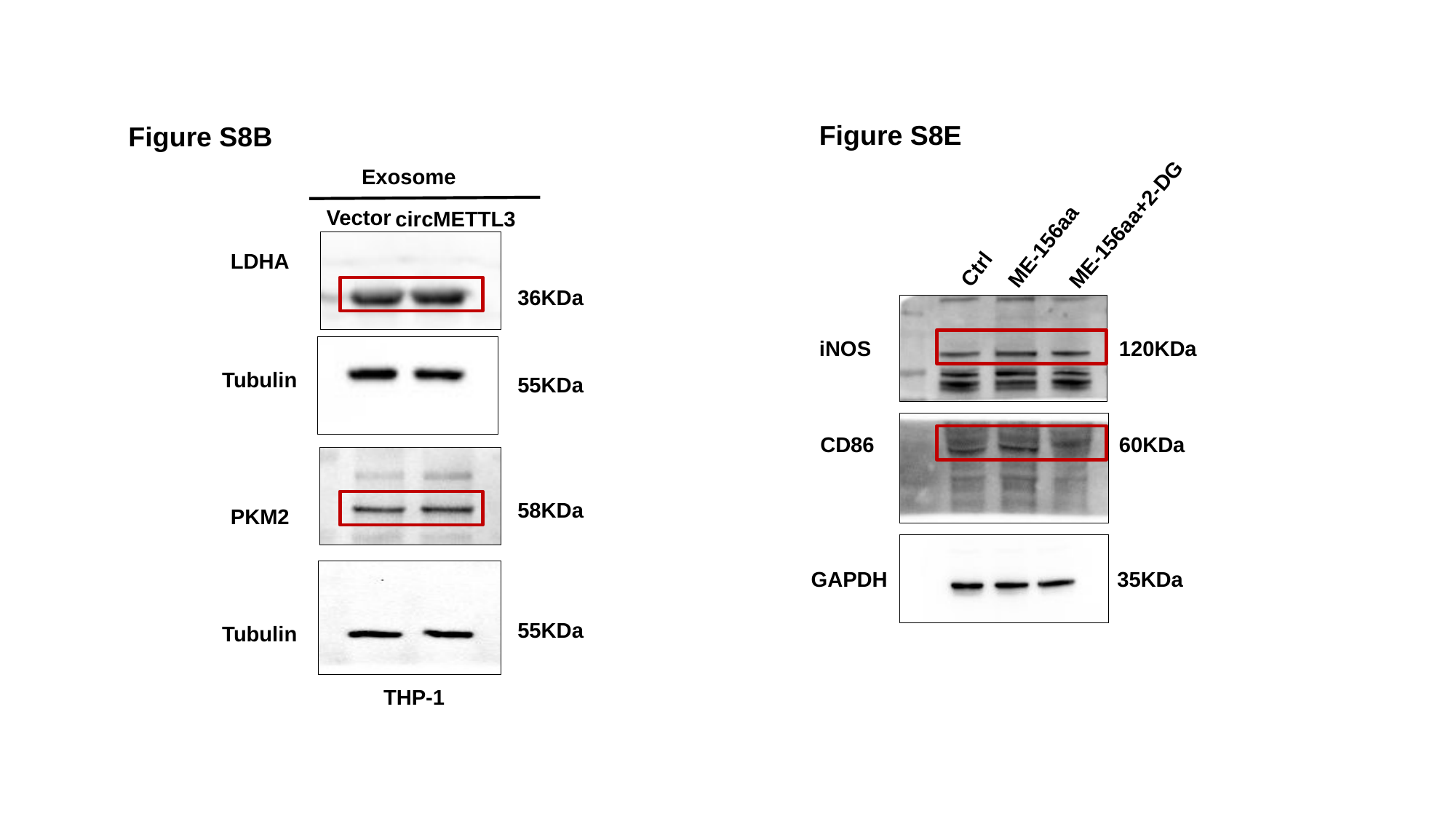

Figure S8E
ME-156aa+2-DG
 ME-156aa
Ctrl
iNOS
CD86
GAPDH
120KDa
60KDa
35KDa
Figure S8B
Exosome
Vector
circMETTL3
LDHA
36KDa
Tubulin
55KDa
58KDa
PKM2
55KDa
Tubulin
THP-1

## Slide 9
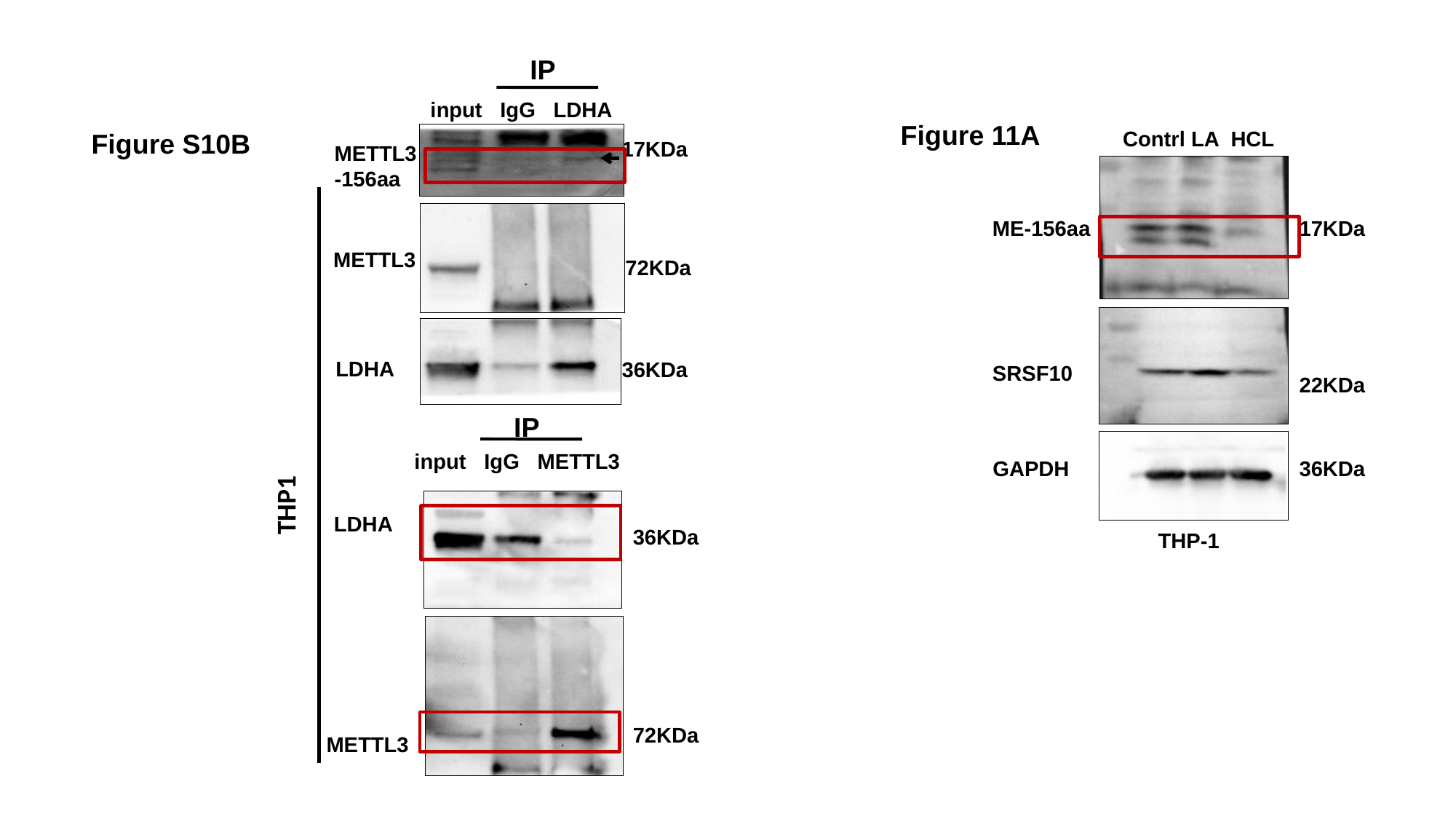

IP
input IgG LDHA
Figure S10B
17KDa
METTL3-156aa
METTL3
72KDa
LDHA
36KDa
IP
input IgG METTL3
THP1
LDHA
36KDa
72KDa
METTL3
Figure 11A
Contrl LA HCL
ME-156aa
17KDa
SRSF10
22KDa
GAPDH
36KDa
THP-1

## Slide 10
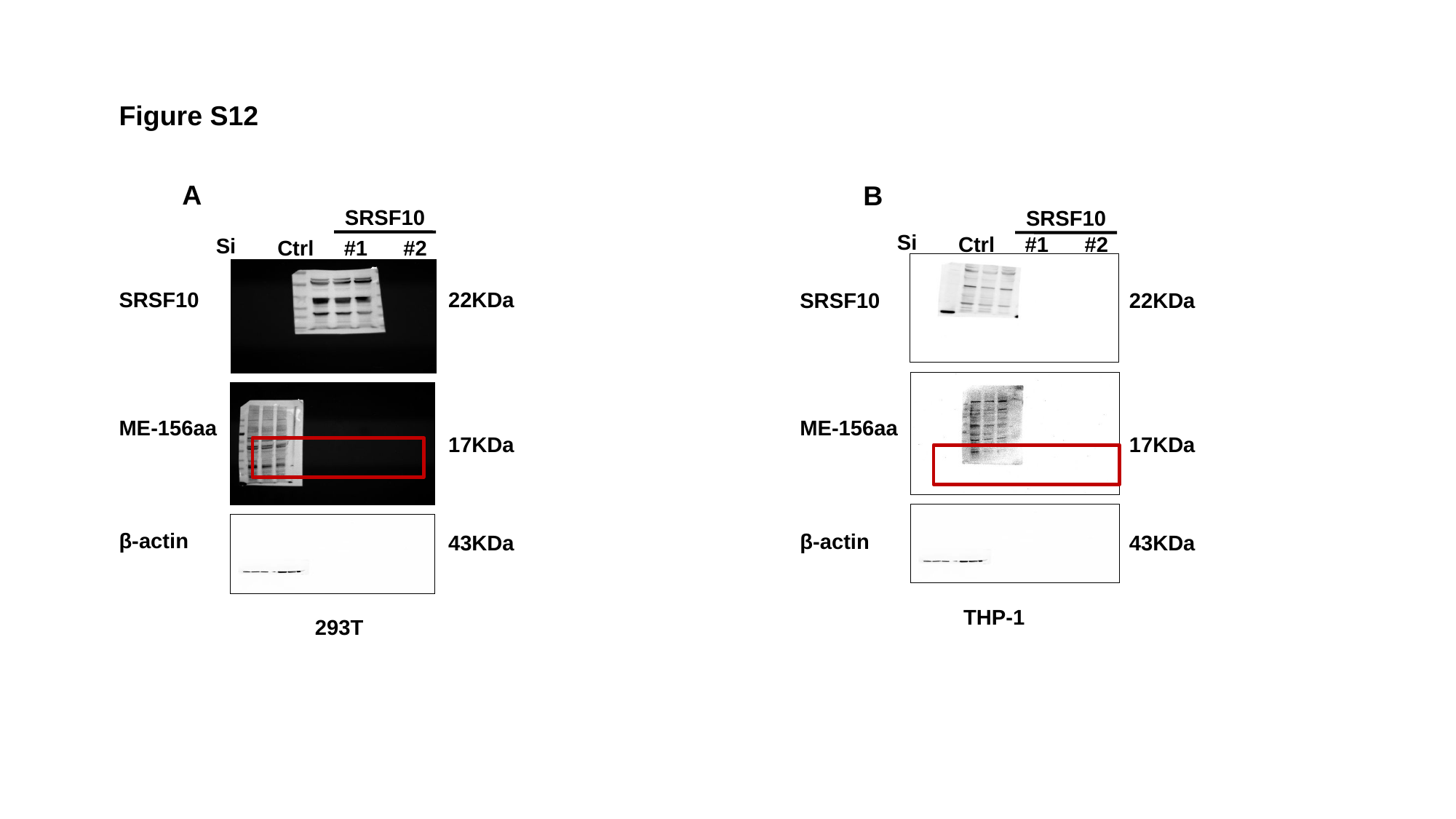

Figure S12
A
SRSF10
Si
Ctrl #1 #2
22KDa
SRSF10
ME-156aa
17KDa
β-actin
43KDa
293T
B
SRSF10
Si
Ctrl #1 #2
22KDa
SRSF10
ME-156aa
17KDa
β-actin
43KDa
THP-1
